# Phytochemical composition, antimicrobial activities, and cholinesterase inhibitory properties of the lichen *Usnea diffracta* Vain

**DOI:** 10.3389/fchem.2022.1063645

**Published:** 2023-01-06

**Authors:** Yi-Meng Hao, Yuan-Cong Yan, Qing Zhang, Bing-Qian Liu, Chang-Sheng Wu, Li-Ning Wang

**Affiliations:** ^1^ School of Chinese Materia Medica, Tianjin University of Traditional Chinese Medicine, Tianjin, China; ^2^ State Key Laboratory of Microbial Technology, Institute of Microbial Technology, Shandong University, Qingdao, China

**Keywords:** *Usnea diffracta* Vain, phenols, dibenzofurans, usnic acid, antimicrobial activities, acetylcholinesterase inhibition

## Abstract

Lichens are important sources of versatile bioactive compounds. Two new dibenzofurans **(1–2)**, a multi-substituted single benzene ring **(3)**, and two organic acid compounds **(4–5)** along with 25 known compounds **(6–30)** were isolated from the lichen *Usnea diffracta* Vain. Their structures were identified by physicochemical properties and spectral analyses. Compounds **1–30** were tested for inhibitory activities against *Staphylococcus aureus, Escherichia coli*, and *Candida albicans* by the disk diffusion method and microdilution assay respectively. Compound **3** showed moderate inhibitory activities against *S. aureus* and *E. coli* with the inhibition zone (IZ) of 6.2 mm and 6.3 mm, respectively. Depside **10** exhibited good activity against *S.aureus* and *C. albicans* with 6.6 mm and 32 μg/ml, respectively. The acetylcholinesterase inhibitory activities of compounds **1, 2,** and **6–8** with the characteristic dibenzofuran scaffold were evaluated var anti-AChE assay and a molecular docking study. Compound **2** could better inhibit AChE at the concentration of 0.3 μmol/ml with a value of 61.07 ± 0.85%. The molecular docking study also demonstrated that compound **2** had the strongest binding affinity among the five dibenzofurans, and the “-CDOCKER Energy” value was 14.4513 kcal/mol.

## 1 Introduction

Lichens are autotrophic symbionts which are composed of mycobiontic fungi (mainly Ascomycetes) and one or more photosynthetic organisms (algae or cyanobacteria). Lichens have a remarkable ability to survive in extreme environmental conditions ranging from deserts to polar areas. Their specific secondary metabolites can protect them from physical and biological attacks (Reddy et al., 2019; Popovici et al., 2022). Lichens are not only ecologically important but also significant medicinal natural product resources, especially phenolic secondary metabolites, including phenols (orinol), dibenzofurans (usnic acid and its derivatives), depsidones (norstictic acid), depsides (diffractaic acid), depsones (picrolichenic acid), and pulvinic acid derivatives (vulpinic acid). There are about 26,000 species of lichens belonging to 500 genera in the world, among which more than 360 *Usnea* species are found (Salgado et al., 2018).

*Usnea diffracta* Vain, belonging to the genus *Usnea*, is widely used as a folk medicine for the treatment of diarrhea, stomachache, ulcer, tuberculosis, pneumonia, malaria, wounds, and parasitic diseases (Singh B. et al., 2016; Zhao et al., 2019). Depsidones, depsides, depsones, quinones, polyphenolics, polysac-charides, and dibenzofurans are the main compounds of *Usnea* (Salgado et al., 2018). Previous studies have showed numerous good pharmacological activities of *U. diffracta*, such as antimicrobial, antioxidant, antitumor, antiviral, anti-inflammatory, cardiovascular protective, anti-genotoxic, and antiproliferative effects (Behera et al., 2012; Lai et al., 2013; Ceker et al., 2015; Koparal, 2015; Nishanth et al., 2015; Singh S. et al., 2016; Vanga et al., 2017; Yang et al., 2017). It is noteworthy that most of these pharmacological activities are attributed to the availability of bioactive depsides and dibenzofuran derivatives (especially usnic acid) (Sun et al., 2016). *Usnea diffracta* and its major constituent usnic acid are potent antimicrobial agents to inhibit growth of different planktonic bacteria and fungi strains (Helena et al., 2012).

Alzheimer’s disease (AD) is a serious degenerative disorder of the central nervous system, characterized by a variety of symptoms, including memory loss, emotional behavior, and cognitive impairment (Singh et al., 2013). One of its pathogeneses mainly includes cholinergic system damage. The cholinergic system of AD patients involves two main cholinesterases (ChE), acetylcholin-esterase (AChE) and butyrylcholinesterase (BChE) (Colovic Mirjana et al., 2013). AChE inhibitors mainly act on the catalytic active site (CAS) at the bottom of AChE, which can relieve the symptoms of mild-to-moderate AD patients (Zhu et al., 2019). At present, cholinesterase inhibitors are mainly selective acetycholinesterase inhibitors (AChEIs) such as tacrine, donepezil, and galantamine (Greig et al., 2005; Furukawa-Hibi et al., 2011; Wu et al., 2016). These drugs have greater side effects or lower response rates in the long-term treatment of patients with mild-to-moderate AD. In order to find natural lead compounds for the treatment of Alzheimer’s disease, benzofurans, as an important pharmacophore, have received extensive attention in the past few years because of their attractive cholinesterase inhibitory activity (Rizzo et al., 2012; Khanam and Shamsuzzaman, 2015; Delogu et al., 2016; Wang et al., 2017). The bibenzofuran structures of usnic acid derivatives are similar to those of galantamine, an anticholinesterase drug for AD. Usnic acid enantiomers show antioxidant and anti-inflammatory activities, which are involved in AD pathogenesis (Araújo et al., 2015; Cazarin et al., 2020).

Hence, in the current research, 30 compounds including five new compounds were isolated and identified from *U. diffracta*. The complete isolation and structural elucidation of all compounds and their antimicrobial activities against two pathogenic bacteria including one Gram-positive strain (*Staphylococcus aureus*) and one Gram-negative bacterial strain (*Escherichia coli*) and fungus strain (*Candida albicans*) were described. Among them, the inhibitory activities on AChE of usnic acid derivatives **1**, **2**, and **6**–**8** were reported. The interaction between these compounds and the active centers of AChE were investigated by molecular docking.

## 2 Results

### 2.1 Isolated compounds from *Usnea diffracta* Vain

**FIGURE 1:**
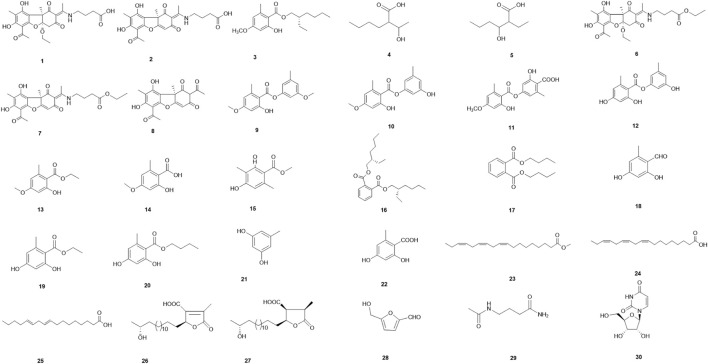
Chemical structures of compounds **1**–**30**.

Thirty compounds (Figure 1) were separated and identified from the ethanol extract of the dry lichen body of *U. diffracta* Vain, including five dibenzofuran compounds (**1**, **2**, and **6**–**8**), eleven multi-substituted single benzene ring compounds (**3** and **13**–**22**) and four depsides compounds (**9**–**12**), four organic acid compounds (**4**, **5**, **24**, and **25**), one organic acid ester compound (**23**), two fatty acids containing furan ring (**26** and **27**), one furfural compound (**28**), one amino acid compound (**29**), and one nucleoside compound (**30**). After searching the SciFinder database, compounds **1**–**5** were identified as new compounds and their structural analysis as follows.

#### 2.1.1 Usenamine G (1)

Pale yellow oil (CH_3_OH). [α]^25^_D_ -132.0° (c 0.05, CH_3_OH); CD (0.59 mg/ml, CH_3_OH) *λ*max (Δε) 304 (−2.19) nm, 278 (+1.74) nm; UV (CH_3_OH) *λ*max (log ε): 223 (3.92) nm, 296 (4.16) nm; IR (KBr): *ν*_max_ 3426, 1639, 1541, 1420, and 1015 cm^−1^; ^1^H NMR (500 MHz, CD_3_OD) and ^13^C NMR (125 MHz, CD_3_OD) data; HR-ESI-MS *m/z* 476.1931 [M + H]^+^ (Calcd. For C_24_H_29_NO_9_, 476.1921) (Table 1; Supplementary Figures S1–S11).

**TABLE 1:** ^1^H and^13^C NMR data (*δ* in ppm, *J* in Hz) for **1**–**5**.

No.	1		2		3		4		5	
	*δ*_H_	*δ*_C_	*δ*_H_	*δ*_C_	*δ*_H_	*δ*_C_	*δ*_H_	*δ*_C_	*δ*_H_	*δ*_C_
1		200		196.2		105.5		179.6		179.2
2		106.8		100.6	11.95 (1H, s)	165.7	2.28 (1H, m)	55.4	2.27 (1H, m)	55.6
3		194.1		188	6.33 (1H, d, *J* = 2.4)	98.8	1.51 (1H, m)	29.6	3.67 (1H, m)	73.0
							1.58 (1H, m)			
4	3.10 (1H, d, *J* = 15.4)	43.7	5.87 (1H, s)	101.4		163.8	1.34 (2H, m)	31.0	1.50 (2H, m)	38.1
	3.15 (1H, d, *J* = 15.4)									
4a		111.6		172						
5a		158.5		154.8						
5					6.28 (1H, d, *J* = 2.4)	111.1	1.34 (2H, m)	23.8	1.38 (2H, m)	19.9
6		102.3		99.9		143.0	0.91 (3H, t, *J* = 6.9)	14.3	0.94 (3H, m)	14.3
7		163.8	13.40 (1H, s)	161.5	2.52 (3H, s)	24.6	3.86 (1H, m)	69.7	1.60 (2H, m)	23.1
8		106.5		105.3		172.2	1.19 (3H, d, *J* = 6.3)	21.3	0.94 (3H, m)	12.4
9		161.2	12.31 (1H, s)	156.7	4.27 (2H, m)	67.6				
9a		107.6		104.2						
9b		61.2		55.3						
10	1.55 (3H, s)	19.2	1.64 (3H, s)	30.7	1.71 (1H, m)	38.8				
11		176.1		174	1.39 (2H, m)	30.6				
12	2.52 (3H, s)	18.0	2.58 (3H, s)	17.1	1.32 (2H, m)	29.0				
13	1.96 (3H, s)	7.4	1.97 (3H, s)	6.5	1.32 (2H, m)	23.0				
14		202.8		199.9	0.90 (3H, t, *J* = 7.0)	14.0				
15	2.55 (3H, s)	31.4	2.63 (3H, s)	30	1.45 (2H, m)	24.0				
16	3.88 (1H, q, *J* = 7.0)	60.7			0.94 (3H, t, *J* = 7.5)	11.1				
	3.87 (1H, q, *J* = 7.0)									
17	1.23 (3H, t, *J* = 7.0)	15.7								
18										
1′	3.59 (2H, t, *J* = 7.3)	44.2	3.54–3.59 (2H, m)	41.8						
2′	1.94–2.01 (2H, m)	25.4	1.86 (2H, m)	23						
3′	2.43 (2H, t, *J* = 7.1)	31.7	2.35 (2H, t, *J* = 7.3)	29.7						
4′		176.2								
5′										
6′										
-OCH_3_					3.80 (3H, s)	55.3				
-NH-			13.05 (1H, t, *J* = 5.2)							
-COOH				172.7						

**FIGURE 2:**
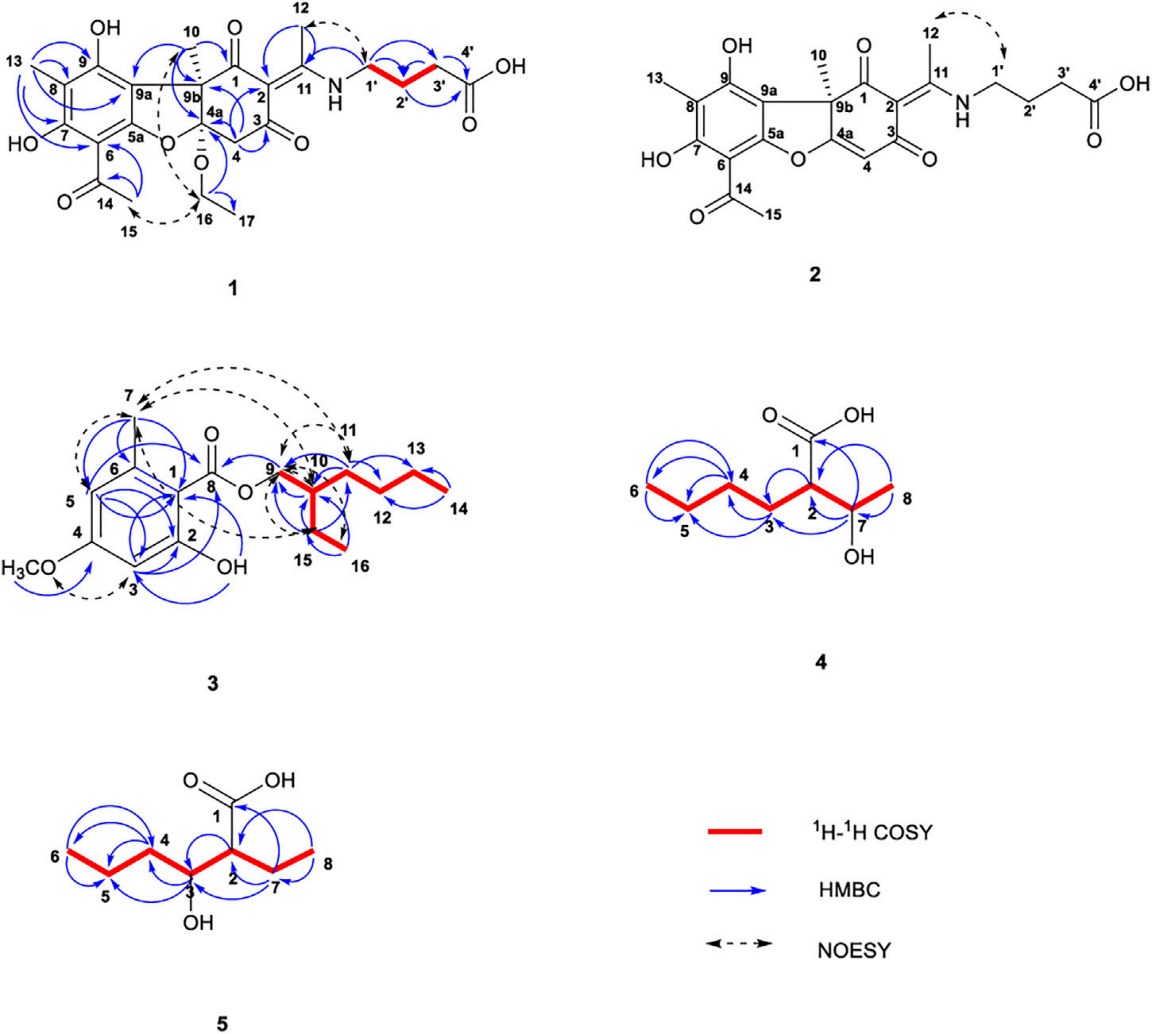
Chemical structures of compounds **1**–**5** along with the key ^1^H-^1^H COSY, HMBC, and NOESY interactions.

The ^1^H NMR data of **1** in CD_3_OD showed signals for five methyl groups: *δ* 1.23 (3H, t), 1.55 (3H, s), 1.96 (3H, s), 2.52 (3H, s), and 2.55 (3H, s); four methylene groups: *δ* 2.43 (2H, t), 1.94–2.01 (2H, m), 3.59 (2H, t), 3.88 (1H, q) and 3.87 (1H, q); two single hydrogen signals: *δ* 3.10 (1H, d) and 3.15 (1H, d). The coupling constants at *δ* 3.10 and 3.15 are both 15.4 Hz, which is speculated to be a carbon coupling hydrogen signal. The ^13^C NMR spectrum showed 24 carbon resonances that consisted of three carbonyl signals (*δ* 202.8, 200.0, and 194.1), aromatic carbon signals and alkene carbon signals (*δ* 161.2, 163.8, 158.5, 107.6, 106.8, 106.5, and 102.3), the chemical shifts 176.2 and 176.1 may contain carboxyl signals, as well as oxycarbon and aliphatic carbon signals. According to the DEPT 135 spectra (Supplementary Figure S11), five methylenes (*δ* 60.7, 44.2, 43.7, 31.7, and 25.4) were existed in compound **1**. The HSQCs (Supplementary Figure S8) indicated directly linked hydrocarbon correlations. Combined with ^1^H-^1^H COSY, the correlations (Figure 2; Supplementary Figure S7) from *δ*_H_ 3.10 to *δ*_H_ 3.15, *δ*_H_ 1.23 to *δ*_H_ 3.88/3.87, and *δ*_H_ 1.97 to *δ*_H_ 2.43/3.59 further verified the existence of -CH_2_-, -OCH_2_CH_3_, and -CH_2_-CH_2_-CH_2_-. The HMBCs (Figure 2; Supplementary Figure S9) from *δ*_H_ 1.55 to *δ*_C_ 200.0/111.6/107.6/61.2 indicated that the angular methyl group was located at C-9b, while those from *δ*_H_ 2.55 to *δ*_C_ 102.3/202.8 suggested that C-14 was connected to C-6. The correlations from *δ*_H_ 2.52 to *δ*_C_ 106.8/176.1 established the connection of C-11 with C-2. The HMBCs from *δ*_H_ 3.88/3.87 to *δ*_C_ 15.7/111.6 suggested the connection of an ethoxy group at C-4a and from *δ*_H_ 2.43/1.94–2.01 to *δ*_C_ 176.2 and *δ*_H_ 3.59 to *δ*_C_ 176.1 revealed the presence of a C-2 enamine moiety in compound **1**. The configuration of the double bond was determined by the NOESY data (Figure 2; Supplementary Figure S10) *δ*_H_ (2.52) - *δ*_H_ (3.59) as *E*, analysis of the correlation between *δ*_H_ (1.55/2.55) and *δ*_H_ (3.88/3.87) dedicated that 9b-CH_3_ and 4a-OCH_2_CH_3_ were on the same side. In addition, the CD spectrum (Supplementary Figure S2) of compound **1** was compared with the related literature previously published (Yu X. et al., 2016). Compound **1** presented one negative maximum at 298 nm and positive at 278 nm, which was consistent with the CD spectrum of Usneamine B, indicating that the absolute configuration of compound **1** was 4a*R*/9b*R*, as represented in Figure 2. Overall, compound **1** was identified as a new compound by SciFinder inquiry, named Usenamine G.

#### 2.1.2 Usenamine H (2)

Pale yellow oil (CH_3_OH). [α]^25^_D_ +104.0° (c 0.05, CH_3_OH); CD (0.59 mg/mL, CH_3_OH) λmax (Δε) 332 (+0.59) nm; ^1^H NMR (600 MHz, DMSO-d_6_) and ^13^C NMR (150 MHz, DMSO-d_6_) data; HR-ESI-MS *m/z* 430.1518 [M + H]^+^ (Calcd. For C_22_H_23_NO_8_, 430.1502) (Table 1; Supplementary Figures S12–S16). A comparison of the ^1^H and ^13^C NMR data of compound **2** with the ^1^H (500 MHz, CD_3_OD) and ^13^C NMR (125 MHz, CD_3_OD) data of **1** revealed their structural similarities, except for the presence of alkene proton signal at *δ*_H_ 5.87 (1H, s)/*δ*_C_ 101.4 (C-4), and the absence of -OCH_2_ - (C-16) and -CH_3_ segments (C-17). The NOESY correlation (Supplementary Figure S16) between H-12 (*δ* 2.58) and H-1′ (*δ* 3.54–3.59) suggested an E-configuration for the double bond and it was the same as compound **1** (Figure 2). The similar sign of the specific rotation [α]^25^_D_ +104.0° (c .05, CH_3_OH) and positive at 333 nm, which was consistent with the CD spectrum of Usneamine A (Yu X. et al., 2016), indicating that the absolute configuration was 9b*R,* as represented in Figure 2. The spectroscopic data were in accord with the literature (Bruno et al., 2013), compound **2** was identified as a new compound by SciFinder inquiry, named Usneamine H.

#### 2.1.3 2-Ethylhexyl-4-methoxy orsellinate (3)

White oil (CH_3_OH). [α]^25^_D_ -12.0° (c 0.05, CH_3_OH); CD (0.09 mg/ml, CH_3_OH) λmax (Δε) 227 (+0.28) nm, 253 (+0.08) nm, 266 (-0.06) nm, 227 (+0.03) nm; UV (CH_3_OH) λmax (log ε): 215 (4.27) nm, 263 (4.03) nm, 301 (3.61) nm; IR (KBr): *ν*_max_ 3462, 2960, 2931, 1653, 1617, 1576, 1456, 1257, 1159 cm^−1^; ^1^H NMR (500 MHz, CDCl_3_) and ^13^C NMR (125 MHz, CDCl_3_) data; HR-ESI-MS m/z 295.1897 [M + H]^+^ (Calcd. For C_17_H_26_O_4_, 295.1909) (Table 1; Supplementary Figures S17–S27).

The ^1^H NMR data of **3** in CDCl_3_ showed signals for a hydroxyl: *δ* 11.95 (1H, s); two m-position hydrogen of benzene ring: *δ* 6.33 (1H, d, *J* = 2.4 Hz) and 6.28 (1H, d, *J* = 2.4 Hz); a -O-CH_2_- fragment: *δ* 4.27 (2H, m); a methoxy group: *δ* 3.80 (3H, s); a methyl attached to the benzene ring: *δ* 2.52 (3H, s); an aliphatic chain: *δ* 1.71 (1H, m), 1.45 (2H, m), 1.39 (2H, m), and 1.32 (4H, m); and two terminal methyl of aliphatic chain: *δ* 0.94 (3H, t, *J* = 7.5 Hz) and 0.90 (3H, t, *J* = 7.0 Hz). The ^13^C NMR spectrum revealed 17 carbon resonances that consisted of a -COO- fragment signal (*δ* 172.2), a -O-CH_2_- fragment signal (*δ* 67.6), a -O-CH_3_ fragment signal (*δ* 55.3), six aromatic carbon signals (*δ* 105.5, 165.7, 98.8, 163.8, 111.1, and 143.0) and eight aliphatic carbon signals (*δ* 38.8, 30.6, 29.0, 24.6, 24.0, 23.0, 14.0, and 11.1). The DEPT spectra (Supplementary Figure S27) demonstrated five methylene signals: *δ* 67.6, 30.6, 29.0, 24.0, and 23.0. The HSQCs (Supplementary Figure S24) indicated directly linked hydrocarbon correlations. According to the HMBCs (Figure 2; Supplementary Figure S25), correlations from *δ*_H_ (0.94) to *δ*_C_ (24.0/38.8), *δ*_H_ (0.90) to *δ*_C_ (23.0/29.0), *δ*_H_ (1.32) to *δ*_C_ (14.0/23.0/29.0/30.6), and *δ*_H_ (1.39) to *δ*_C_ (23.0/29.0) showed the presence of -CH_2_-CH_2_-CH_2_-CH_3_ fragment in the aliphatic chain, and *δ*_H_ (0.94) to *δ*_H_ (1.45) showed -CH_2_-CH_3_. The correlations from *δ*_H_ (1.38) to *δ*_C_ (38.0/67.6), *δ*_H_ (1.45) to *δ*_C_ (67.6/38.8/11.1/30.6), *δ*_H_ (1.71) to *δ*_C_ (67.6/24.0/11.1/30.6), and *δ*_H_ (4.27) to *δ*_C_ (23.0/30.6/38.8/172.7) together indicated the fragment of CH_3_-CH_2_-CH-CH_2_-OOC-, in which -CH- was linked to the butyl group. The *δ*_H_ (2.51)–*δ*_C_ (143.0/111.1/105.5) and *δ*_H_ (3.80)–*δ*_C_ (163.8) revealed that the methyl and oxymethyl existed on the benzene ring. At the same time combined with COSY correlations (Supplementary Figure S23), the planar structure of compound **3** was shown in Figure 2. The main hydrogen-related signals shown by the NOESY spectra (Figure 2; Supplementary Figure S26) were *δ*_H_ (4.27)–*δ*_H_ (0.94/1.32/1.39/1.45/1.71), *δ*_H_ (2.51)–*δ*_H_ (1.32/1.39/1.45/1.71), *δ*_H_ (6.33)–*δ*_H_ (3.80), and *δ*_H_ (6.28)–*δ*_H_ (2.52). In addition, the CD spectrum (Supplementary Figure S18) of compound **3** was compared with the related literature previously published (Nahrstedt et al., 1990). Compound **3** presented one negative which was consistent with (*S*)-2-Methylbutan-1-yl-*β*-D-glucopyranoside, indicating that the absolute configuration of C-10 was *S*. Through SciFinder database query, it was confirmed that compound **3** was a new compound and shared the similar structure with 2-ethylhexyl orsellinate (Bui et al., 2020), named 2-ethylhexyl-4-methoxy orsellinate.

#### 2.1.4 2-(1-Hydroethyl)-hexanoic acid (4)

White oil (CH_3_OH). [α]^25^_D_ -8.0° (c 0.07, CH_3_OH); CD (0.56 mg/mLl CH_3_OH) λmax (Δε) 199 (-0.22) nm; UV (CH_3_OH) λmax (log ε): 202 (2.68) nm, 222 (2.35) nm, 274 (1.70) nm; IR (KBr): *ν*_max_ 3416, 2958, 2932, 1694, 1403, 1204, and 1119 cm^−1^; ^1^H NMR (600 MHz, CD_3_OD) and ^13^C NMR (150 MHz, CD_3_OD) data; HR-ESI-MS *m/z* 159.1038 [M–H]^-^ (Calcd. For C_8_H_16_O_3_, 159.1021) (Table 1; Supplementary Figures S28–S38).

The ^1^H NMR data of compound **4** in CD_3_OD showed signals for 14 hydrogen atoms. According to the chemical shift, 1.19 (3H, d, *J* = 6.3 Hz), it is inferred that there is a -CH-CH_3_ fragment in the structure, and the shift 0.91 (3H, t, *J* = 6.9 Hz) showed the existence of -CH_2-_CH_3_. The ^13^C NMR spectrum showed eight carbon resonances that consisted of one carbonyl signals (*δ* 179.6), one -O-C- fragment signal (*δ* 69.7), one -O-CH_3_ fragment signal (δ 55.4), and five aliphatic carbon signals (*δ* 31.0, 29.6, 23.8, 21.3, and 14.3). According to the DEPT 135 spectra (Supplementary Figure S38), three methylenes (*δ* 31.0, 29.6, and 23.8) were existed in compound **4**. The HSQC spectrum (Supplementary Figure S35) revealed directly linked hydrocarbon correlations. Correlations presented in ^1^H-^1^H COSY (Figure 2; Supplementary Figure S34) from *δ*_H_ (1.19) to *δ*_H_ (3.86), *δ*_H_ (3.86) to *δ*_H_ (2.28), *δ*_H_ (1.51/1.58) to *δ*_H_ (1.34/2.28), and *δ*_H_ (1.34) to *δ*_H_ (.91) further verified the existence of -CH-CH_3_ and -CH_2_-CH_2_-CH_3_. The HMBCs (Figure 2; Supplementary Figure S36) from *δ*_H_ (.91/1.51/1.58) to *δ*_C_ (23.8/31.0) and *δ*_H_ (1.34) to *δ*_C_ (14.3/23.8/31.0) indicated the presence of CH_3_-CH_2_-CH_2_-CH_2_-. The correlations from *δ*_H_ (2.28) to *δ*_C_ (21.3/29.6/69.7) and *δ*_H_ (3.86) to *δ*_C_ (29.6/55.4/179.6) showed the fragment of COOH-CH-CH-OH. The signal of *δ*_H_ (1.19) - *δ*_C_ (55.4/69.7) confirmed that the terminal methyl (C-8) was connected to C-7. The main hydrogen-related signals shown by the NOESY spectra (Supplementary Figure S37) were *δ*_H_ (2.28) - *δ*_H_ (3.86/1.51/1.58/1.34/1.21) and *δ*_H_ (3.86) - *δ*_H_ (1.51/1.58/1.21). The structure and spectroscopic data were shown in Figure 2 and Table 1. Overall, compound **4** was identified as a new compound by SciFinder inquiry, named 2-(1-hydroethyl)-hexanoic acid. Its configuration needs to be further determined.

#### 2.1.5 2-Ethyl-3-hydroxyhexanoic acid (5)

White oil (CH_3_OH). [α]^25^_D_ -8.0° (c 0.05, CH_3_OH); ^1^H NMR (600 MHz, CD_3_OD) and ^13^C NMR (150 MHz, CD_3_OD) data; HR-ESI-MS *m/z* 159.0546 [M–H]^-^ (Calcd. For C_8_H_16_O_3_, 159.1021) (Table 1; Supplementary Figures S39–S46).

A comparison of the ^1^H and ^13^C NMR data of compound **5** and compound **4** revealed their structural similarities, except for the presence of two proton signals at *δ*_H_ 1.60 (2H, m)/*δ*_C_ 23.1 (C-7) and one proton signal at *δ*_H_ 3.67 (1H, m)/*δ*_C_ 73.0 (C-3). According to the chemical shift, it is inferred that the hydroxyl group (C-7) of compound **4** was connected to the C-3 in compound **5**. Also, the ^1^H NMR data of compound **5** showed signals for 14 hydrogen and the ^13^C NMR spectrum showed eight carbon resonances. According to the DEPT 135 spectra (Supplementary Figure S46), three methylenes were existed in compound **5**. The HSQC spectrum (Supplementary Figure S43) revealed directly linked hydrocarbon correlations. Correlations presented in ^1^H-^1^H COSY (Supplementary Figure S42) from *δ*_H_ (1.50) to *δ*_H_ (3.67) and *δ*_H_ (1.60) to *δ*_H_ (0.94) further verified the existence of -CH_2_-CH_2_-CH-OH and -CH_2_-CH_3_. The relations of *δ*_H_ (0.94)–(1.60/3.67) showed that the -CH-COOH fragment was linked to -CH-OH and -CH_2_-CH_3_ fragment and *δ*_H_ (0.94)–(1.38) indicated that the terminal methyl was connected to the -CH_2_-CH_2_-. The correlations of H-2/C-1, 3, 4, 7, 8; H-3/C-1, 2, 4, 5; H-4/C-3, 5, 6; H-5/C-3, 4, 6; H-6/C-4, 5; H-7/C-1, 2, 3, 4, 8; and H-8/C-2, 7 were observed in HMBC (Supplementary Figure S44) and H-2/H-4, 7, 3 and H-6/H-4, 7, 5 in NOESY (Supplementary Figure S45). The structure and spectroscopic data were shown in Figure 2; Table 1. The configuration of the two chiral carbons could not be determined. Compound **5** was identified as a new compound by SciFinder inquiry, named 2-ethyl-3-hydroxyhexanoic acid (Wahl et al., 2001).

The known compounds isolated and identified by spectroscopic techniques were Usneamine F (**6**) (Yu X. et al., 2016), Usneamine E (**7**) (Yu X. et al., 2016), (+)-usnic acid (**8**) (Laxi et al., 2013), 2′-*O*-methylevernol (**9**) (Huneck et al., 1989), 3-hydroxy-5-methylphenyl-2-hydroxy-4-methoxy-6-methylbenzoate (**10**) (Yu X.L. et al., 2016), evernic acid (**11**) (Tang et al., 2015), lecanorin (**12**) (Bui et al., 2020), ethyl everninate (**13**) (Yu X.L. et al., 2016), evernesic acid (**14**) (Nishitoba et al., 1987), methyl-2,4-dihydroxy-3,6-dimethylbenzoate (**15**) (Feng et al., 2009), bis-(2*S*-ethylhexyl)benzene-1,2-dicarboxylate (**16**) (Su et al., 2009), dibutyl phthalate (**17**) (Li et al., 2009), orsellinaldehyde (**18**) (Zheng et al., 2012), ethyl orsellinate (**19**) (Tuan et al., 2020), *n*-butyl orsellinate (**20**) (Li et al., 2006), orcinol (**21**) (Eliwa et al., 2019), orsellinic acid (**22**) (Wang et al., 2009), *α*-linolenic acid methyl ester (**23**) (Zou et al., 2021), *α*-linolenic acid (**24**) (Qin et al., 2010), linoleic acid (**25**) (Zhao et al., 2020), constipatic acid (**26**) (Chester and Elix, 1979), 18*R*-hydroxy-dihydroalloprotolichesterinic acid (**27**) (Rezanka and Guschina, 2001), 5-hydromethyl furaldehyde (**28**) (Feng et al., 2011), 4-(acylamino)butyramides (**29**) (Graham et al., 1987), and uridine (**30**) (Gu et al., 2017); (Figure 1).

### 2.2 Antimicrobial activity

In this study, the antimicrobial activities of the isolated compounds were studied *in vitro*. The inhibition zone of the compounds against *S. aureus* and *E. coli* were determined by the disk diffusion method and the values of minimal inhibitory concentration (MIC) against *C. albicans* by microdilution assay. The results (Table 2) revealed that when the amount of compound was 3.2 μg/disk, the inhibition zone (IZ) of compounds **3**, **9**, **10**, and **18** were 6.2, 6.4, 6.6, and 6.6 mm, respectively, and others had no effect in the preliminary screening experiment of anti-*S. aureus*. Among them, **10** and **18** exhibited better antibacterial activities against *S. aureus* than other compounds, but lower than the positive control gentamicin sulfate. In addition, compounds **3** and **30** can better inhibit *E. coli* with IZ of 6.3 mm than other compounds at 3.2 μg/disk. The assay of anti-*C. albicans* showed that the MIC value of tested compound **10** was 32 μg/ml, which exhibited the strongest inhibitory activity among all compounds. In conclusion, the results of the preliminary screening demonstrated that compound **3** had certain inhibitory activities against *S. aureus* and *E. coli* but no anti-*C. albicans* activity at 64 μg/ml, compound **9** had certain inhibitory effect on *S. aureus* and *C. albicans* with MIC of 64 μg/ml. Furthermore, in this preliminary screening, compound **10** demonstrated the strongest inhibition against *S. aureus* and *C. albicans* among all compounds.

**TABLE 2:** Antimicrobial activities of compounds from *U. diffracta* Vain. (The IZ value indicated the diameter of the inhibition zone at 3.2 μg/disk. The MIC value indicated the minimal inhibitory concentration. “—” indicated no inhibitory activity in 3.2 μg/disk or 64 μg/ml.)

No.	IZ (mm)/*S. aureus*	No.	IZ (mm)/*E. coli*	No.	MIC(μg/ml)/*C. albicans*
**1**	—	**1**	—	**1**	64
**2**	—	**2**	—	**2**	64
**3**	6.2	**3**	6.3	**3**	—
**4–8**	—	**4**	—	**4–5**	No antifungal assay
**9**	6.4	**5**	—	**6–9**	64
**10**	6.6	**6**	—	**10**	32
**11–17**	—	**7**	—	**11**	—
**18**	6.6	**8**	—	**12–15**	64
**19**	—	**9**	—	**16**	—
**20**	—	**10**	—	**17**	64
**21**	—	**11–29**	—	**18–25**	—
**22–30**	—	**30**	6.3	**26–27**	64
**DMSO**	—	**DMSO**	—	**28**	—
**Gentamicin sulfate**	12.3	**Gentamicin sulfate**	12.4	**29–30**	64

### 2.3 Acetylcholinesterase inhibitory activity

The activities of five dibenzofurans [Usneamine G (**1**), Usenamine H (**2**), Usneamine F (**6**), Usneamine E (**7**), and (+)-usnic acid (**8**)] against AChE were evaluated in the current work. Compound **2** (at the concentration of 0.3 μmol/ml) exhibited the strongest AChE inhibitory activity among the five compounds, with the inhibition value of 67.56 ± 0.49% (as shown in Table 3). Others also exhibited moderate AChE inhibition at the concentration of 0.3 μmol/ml with values of 61.18 ± 1.16%, 61.07 ± 0.85%, 57.61 ± 0.23%, and 58.06 ± 0.73%, respectively.

**TABLE 3:** AChE inhibitory activities of five dibenzofurans from *U. diffracta* Vain.

Compound	AChE inhibition (% at 0.3 μmol/ml)
**1**	61.18 ± 1.16
**2**	67.56 ± 0.49
**6**	61.07 ± 0.85
**7**	57.61 ± 0.23
**8**	58.06 ± 0.73

### 2.4 Molecular docking study

In order to study the binding affinity related to the inhibition of acetylcholinesterase, five compounds of dibenzofurans: Usneamine G (**1**), Usenamine H (**2**), Usneamine F (**6**), Usneamine E (**7**), and (+)-usnic acid (**8**) were simulated. The results of molecular docking revealed that the five compounds had good binding affinities with the active pockets of AChE, and the ‘-CDOCKER Energy’ values were 1.01935 kcal/mol, 14.4513 kcal/mol, -1.95894 kcal/mol, 12.8461 kcal/mol, and -3.65539 kcal/mol, respectively, higher than that of the original ligand galantamine (-9.06147 kcal/mol). Among them, compound **2** exhibited the highest AChE inhibition, which was consistent with the previous work. The interactions data for compounds **1**, **2**, and **6**–**8** docked into the active site gorge of AChE is shown in Table 4.

**TABLE 4:** Binding interaction data of compounds **1**, **2**, and **6**–**8** docked into the active site gorge of AChE.

Ligand	-CDOCKER Energy (kcal/mol)	Interacting site	Residue	Type of interaction	Distance	Ligand interacting moiety
					(Å)	
**1**	1.01935	Anionic subsite	Tyr133	H-bonded	2.12	C7-OH
		Anionic subsite	Tyr133	H-bonded	2.45	C14-C=O
			Val294	H-bonded	2.41	C4′-C=O
		Oxyanion hole	Gly120	H-bonded	2.82	C14-C=O
		Oxyanion hole	Gly120	Amide–Pi stacked	4.1	Benzene ring
		PAS	Tyr124	H-bonded	2.34	C1′-H
		PAS	Tyr124	H-bonded	2.49	C1′-H
		PAS	Tyr124	Pi–alkyl	4.49	C10
		PAS	Tyr341	Pi–cation	3.57	N
		PAS	Asp74	Attractive charge	5.58	N
		CAS	His447	Pi–alkyl	4.92	C17
		Anionic subsite	Tyr337	Pi–alkyl	4.09	C17
		Anionic subsite	Trp86	Pi–alkyl	5.44	C17
**2**	14.4513	Anionic subsite	Tyr133	H-bonded	2.33	C7-OH
		Oxyanion hole	Gly120	Amide–Pi stacked	4.02	Benzene ring
		PAS	Tyr124	H-bonded	2.45	C2′-H
		PAS	Tyr124	H-bonded	2.35	C2′-H
		Acyl pocket	Phe295	H-bonded	2.08	C4′-COOH
		PAS	Tyr341	Pi–cation	3.68	N
		Anionic subsite	Tyr337	Pi–alkyl	4.3	C10
		Anionic subsite	Trp86	Pi–alkyl	4.37	C10
**6**	−1.95894	Anionic subsite	Tyr133	H-bonded	2.28	C7-OH
		PAS	Tyr124	H-bonded	2.04	-NH-
		PAS	Tyr124	Pi–alkyl	5.2	C17
		PAS	Tyr341	H-bonded	2.57	C3-C=O
		PAS	Tyr341	Pi–cation	4.21	N
			Ser125	H-bonded	2.36	C16-H
			Ser125	H-bonded	2.2	C16-H
		Anionic subsite	Trp86	Pi–alkyl	4.28	C10
		Anionic subsite	Tyr337	Pi–alkyl	4.29	C10
		Oxyanion hole	Gly120	Amide–Pi stacked	4	Benzene ring
**7**	12.8461		Gly126	H-bonded	2.51	C4′-C=O
			Ser125	H-bonded	2.68	C4′-C=O
		Anionic subsite	Trp86	Pi–lone pair	2.88	C3-C=O
		Anionic subsite	Trp86	Pi–cation	4.54	N
		Anionic subsite	Glu202	Attractive charge	4.33	N
		PAS	Tyr124	Pi–lone pair	2.86	Benzene ring
		PAS	Tyr124	Pi–alkyl	4.34	C10
		PAS	Tyr341	Pi–Pi	4.67	Benzene ring
		Anionic subsite	Tyr337	Pi–Pi	5.73	Benzene ring
		Anionic subsite	Phe338	Pi–alkyl	4.99	C16
		Acyl pocket	Phe297	Pi–alkyl	4.78	C16
**8**	−3.65539	Oxyanion hole	Gly121	H-bonded	1.83	C14-C=O
		Oxyanion hole	Gly122	H-bonded	1.95	C14-C=O
		CAS	Ser203	H-bonded	2.24	C14-C=O
		Oxyanion hole	Ala204	H-bonded	2.14	C14-C=O
		Oxyanion hole	Gly120	H-bonded	2.68	C3-C=O
		CAS	His447	H-bonded	2.59	C1-C=O
		PAS	Tyr124	Pi–alkyl	5.86	Benzene ring
		PAS	Tyr341	Pi–alkyl	4.02	C16
		Anionic subsite	Tyr337	Pi–alkyl	3.99	C13
		Anionic subsite	Trp86	Pi–alkyl	4.87	C13

**FIGURE 3:**
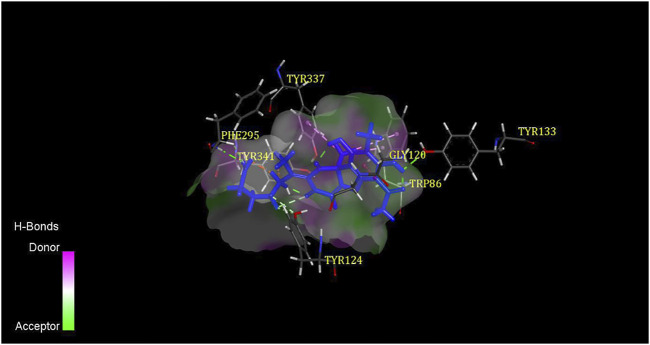
3D binding mode of compound **2** with AChE.

**FIGURE 4:**
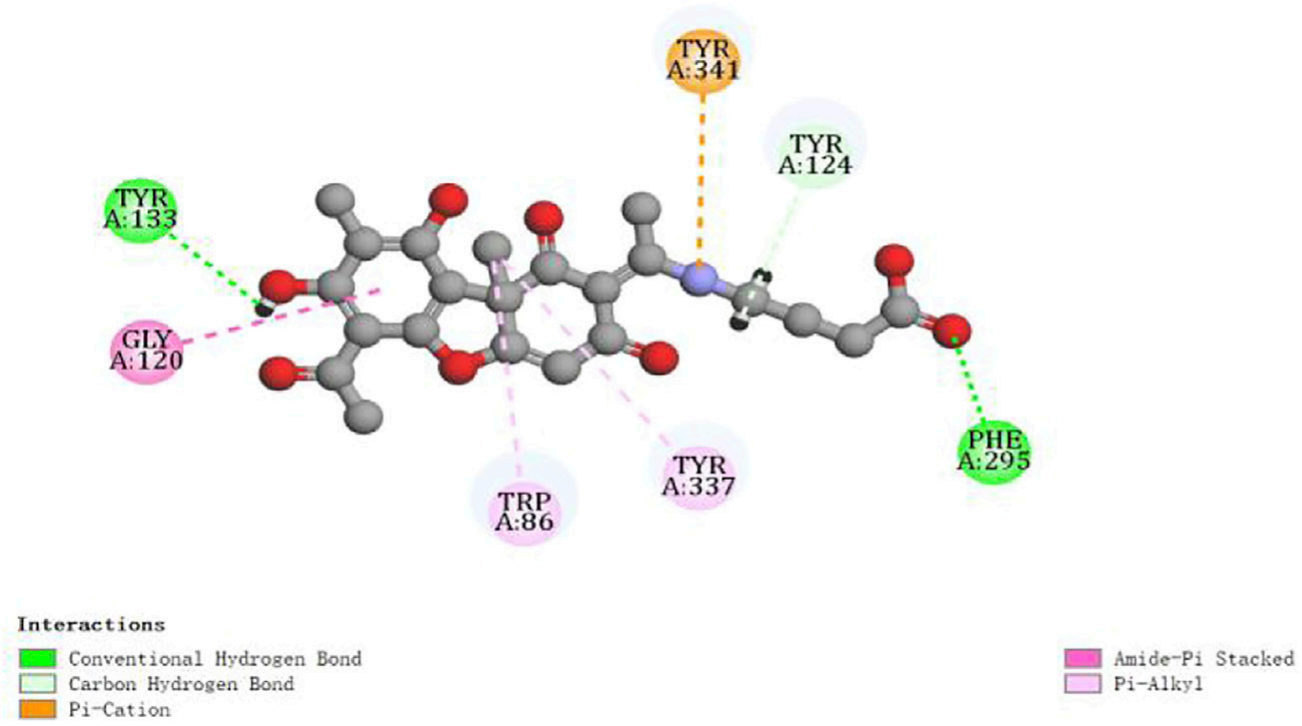
2D diagram of the ligand–protein interaction for compound **2** with AChE.

Regarding AChE, the higher of ‘-CDOCKER Energy’ meant interactions between the five compounds and the 4EY6 were stronger than galantamine. Hydrogen bonding, attractive charge, amide–Pi stacked, Pi–Pi, Pi–alkyl, Pi–cation, Pi–anion, Pi–lone pair interactions were found to be involved between the compounds and the active site residues Tyr133, Tyr337, Trp86, Glu202, and Phe338 in the anionic subsite; Tyr124, Tyr341, and Asp74 in the peripheral active site (PAS); His447 and Ser203 in the catalytic active site (CAS); Gly120, Gly121, Gly122, and Ala204 in the oxyanion hole; and Phe297 and Phe295 in the acyl pocket (Namdaung et al., 2018; Saenkham et al., 2020). Compound **2** was found with the highest AChE (PDB: 4EY6) inhibition, which showed eight tight bindings to key residues (Table 4). The 3D and 2D molecular interaction of compound **2** in the binding pocket of AChE are clearly explained in Figure 3 and Figure 4. The results suggested that compound **2** formed four hydrogen bondings to key residues, namely, two hydrogen atoms of C2’ to Tyr124 (2.45 Å and 2.35 Å) at PAS, C4′-COOH to Phe295 (2.08 Å) at acyl pocket, C7-OH to Tyr133 (2.33 Å) at anionic subsite. Secondly, the benzene ring interacted with the Gly120 (4.02 Å) of the oxyanion hole to form amide–Pi stacked, and C10 interacted with Tyr337 (4.30 Å) and Tyr86 (4.37 Å) at anionic subsite to form Pi–alkyl interaction. In addition, the Pi–catinon interaction between the N atom and the Tyr341 (3.68 Å) located in PAS can also be observed. These interactions played a vital role for anti-AChE activity and may be useful to further explain the inhibition exhibited by compound **2**.

## 3 Discussion

*Usnea* is a kind of lichen traditional Chinese medicine with high-medicinal value, distributed in polar and tropical regions, and widely distributed in Sichuan, Inner Mongolia, Yunnan, Heilongjiang, and other places in China. There are more than 10 common species, such as *U. diffracta* Vain, *U. cavernosa* Tuck, and *U. longissima* Ach, among which *U. longissima* Ach is a large species studied widely (Salgado et al., 2018; Ullah et al., 2019). The phytochemistry of the *Usnea* genus show that it contains more than 60 compounds, which mainly belong to depsides, depsidones, depsones, lactones, quinones, phenolics, polysaccharides, fatty acids, and dibenzofurans (Salgado et al., 2018). Among them, depsides, depsidones, depsones, and dibenzofurans are the unique phenolic components of lichens. These representative aromatic products are compounds derived from the acetyl-polymalonyl pathway, the most characteristic being formed by the bonding of two or three orcinol or 3-orcinol-type phenolic units through ester, ether, and carbon–carbon linkages (Shukla et al., 2010).

The purpose of this study was to determine the chemical constituents from the dry lichen body of *Usnea diffracta* Vain. Following the isolation procedure in this article, we isolated 30 compounds including 16 phenols from *U. diffracta* Vain, which specifically belonged to dibenzofurans, multi-substituted single benzene ring, depsides, organic acid, organic acid ester, fatty acids containing furan ring, furfural, amino acid, and nucleoside compound. After searching the SciFinder database, compounds **1**–**5** were identified as new compounds. Most of the compounds were multi-substituted single benzene ring, dibenzofurans, and depsides, all of which were recognized as the main secondary metabolites of *Usnea*.

*Usnea* lichen has a long history of antimicrobial use as a folk medicine. Modern scientific research reveals that its crude extracts and monomer compounds have good progress in antimicrobial activities. Derivatives of depsides and dibenzofurans are the main resources of its antimicrobial activities, especially the usnic acid is the most prominent which has a relatively wide antimicrobial spectrum. Several studies have confirmed that usnic acid can inhibit growth of different planctonic bacteria and fungi strains, such as *Klebsiella pneumoniae*, *Bacillus subtilis*, *Bacillus cereus*, *Bacillus mycoides*, *Staphylococcus aureus*, and *Escherichia coli* (Sultana and Afolayan, 2011; Helena et al., 2012; Rankovic et al., 2012). The antimicrobial activity of *Usnea diffracta* Vain ethyl acetate extracts against *Actinomyces viscosus* and *Streptococcus mutans* assays showed that ethyl acetate extracts were resistant to these strains (Qi et al., 2009). In this study, the disk diffusion method and the microdilution method were designed to screen the activities of the isolated compounds against *Staphylococcus aureus*, *Escherichia coli*, and *Candida albicans*. An anti-*C. albicans* assay was not performed due to the insufficient amount of compounds **4**–**5**. The results exhibited that compound **3** had certain inhibitory activities against *S. aureus* and *E. coli* but no anti-*C. albicans* activity at 64 μg/ml, compound **9** had certain an inhibitory effect on *S. aureus* and *C. albicans* with the MIC of 64 μg/ml, and compound **10** demonstrated the strongest inhibition against *S. aureus* and *C. albicans* among all compounds. Among them, compounds **9** and **10** belong to depsides. Although five dibenzofurans (**1**, **2**, and **6**–**8**) have no antibacterial effect, they can inhibit the growth of *C. albicans* with the MIC of 64 μg/ml. This further confirmed that derivatives of depsides and dibenzofurans were the main resources of its antimicrobial activities. The result that (+)-usnic acid (**8**) studied in this study showed no activity against *S. aureus* and *E. coli* does not mean that it is inconsistent with previous studies, but can only demonstrate that there is no bacteriostatic effect at this concentration. As this antimicrobial activity assay is only conducted in a certain concentration range for primary activity screening, and cannot be concluded that other compounds have no effect, the follow-up needs to be more detailed and in-depth study of the antimicrobial activities of these compounds.

Since benzofuran has been reported to be a pharmacophore with cholinesterase inhibitory activity, and dibenzofurans, such as (+)-usnic acid, are similar to galantamine in structure, an anticholinesterase drug for AD. We designed experiments to observe the cholinesterase inhibitory activity of the isolated dibenzofurans **1**, **2**, and **6**–**8** and to study whether they can be potential cholinesterase inhibitors or drugs for the treatment of AD. The assay of AChE inhibitory activity was determined by the improved spectrophotometric Ellman’s method (Zhu et al., 2019; Saenkham et al., 2020). Results demonstrated that the five compounds also exhibited AChE inhibition at the concentration of 0.3 μmol/ml with values of 1.01935 kcal/mol, 14.4513 kcal/mol, −1.95894 kcal/mol, 12.8461 kcal/mol, and -3.65539 kcal/mol, respectively. To follow up our results and study the binding affinity related to the inhibition of acetylcholinesterase, molecular docking simulations were performed. The 3D crystal structure of recombinant human AChE complexed with galantamine (code ID: 4EY6) was obtained from the protein data bank (PDB) with a resolution of 2.4 Å and docked with the 3D structure of five compounds (Cheung et al., 2012). Docking analysis exhibited that the investigated compounds had a high affinity for the acetylcholinesterase’s active sites and higher ‘–CDOCKER Energy’ than the original ligand molecule ranged from -3.65539 kcal/mol ((+)-usnic acid) to 14.4513 kcal/mol (Usneamine H) for the target enzyme. Usneamine H exhibited the highest AChE inhibition, which was consistent with the assay of AChE inhibitory activity. It formed four hydrogen bonding, amide–Pi stacked, Pi–catinon interaction, two Pi–alkyl interactions to key residues at PAS, acyl pocket, anionic subsite, and oxyanion hole of AChE. These previous interactions assisted in the understanding of anti-AChE activity. However, considering the simplicity of our anti-acetylcholinesterase activity experiments, whether the isolated compounds can become potential cholinesterase inhibitors still needs further research, such as cell assays and *in vivo* assays.

## 4 Materials and methods

### 4.1 Plant collection

The dry lichens of *Usnea diffracta* Vain were collected from the Anguo traditional Chinese medicine market in Baoding (Hebei, China) in September 2018, and were identified by Professor Lijuan Zhang of Tianjin University of Traditional Chinese Medicine. The voucher specimen (UD-2018-C412-1) has been deposited at the College of Traditional Chinese Medicine, Tianjin University of Traditional Chinese Medicine.

### 4.2 Chemicals and reagents

Chromatographic grade methanol and acetonitrile were obtained from (Concord Technology (Tianjin) Co., Ltd.). Analytical grade petroleum ether, dichloromethane, ethyl acetate, methanol, trifluoroacetic acid, formic acid, and dimethyl sulfoxide were obtained from (Concord Technology (Tianjin) Co., Ltd.). 5,5-dithiobis [2-nitrobenzoic acid] (DTNB, CAS: 69-78-3), acetylthiocholine iodide (ATCI, CAS: 2260-50-6), and acetylcholinesterase (CAS: 9000-81-1) from *Electrophorus electricus* (Sigma Aldrich). Brain–Heart Infusion Agar and Broth were obtained from Qingdao Hope Bio-Technology Co., Ltd. Gentamicin sulfate (CAS: 1405-41-0) was obtained from Shanghai Aladdin Biochemical Technology Co., Ltd.

### 4.3 General

The CD spectrum was recorded by a J-1500 circular dichroic spectrometer (JASCO Co., Japan). NMR spectroscopy was performed on a Bruker AVANCE Ⅲ NMR spectrometer operating at 600 MHz for (^1^H), 150 MHz (^13^C) or Bruker AVANCE Ⅲ 500 NMR spectrometer (Bruker Co., Switzerland). The deuterated solvents used were DMSO-d_6_ and CD_3_OD and the chemical shifts were recorded in ppm. UV spectra were taken on a UV-6150S dual-beam UV-vis spectrophotometer (Shanghai MADAPA Instruments Co., Ltd.). The IR spectrum was detected using a Bruker ALPHA IR spectrophotometer (Bruker Co., Switzerland.). Specific optical rotations were measured using an AUTOPOLV polarimeter (Rudolph Co., United States). The relative molecular mass was measured on a Waters Xevo G2-SUPLC-Q/TOF spectrometer (Waters Co., United States). The samples were prepared by a LC-20AR system, LC-20AP system (Shimadzu, Kyoto, Japan), and Waters 2535 system (Waters Co., United States). Column chromatography included silica gel (200–300 and 300–400 mesh, Qingdao Marine Chemical Co., Qingdao, China), ODS (5–50 μm, YMC Co., Japan), YMC-pack-A (5 μm, 10 × 250 mm, YMC Co., Japan), Shim Pack PRC (ODS 15 μm, 20 × 250 mm, Japan), Sephadex LH-20 (Pharmacia, Sweden), and Macroreticular absorbing resin AB-8 (Tianjin Saizhiwei Technology Co., Tianjin, China). TLC was performed on TLC plates pre-coated with silica gel GF-254 (Qingdao Marine Chemical Co., Qingdao, China). The antimicrobial activities were measured by an HJH-C1112B clean bench (Shanghai Zhicheng Analytical Instrument Manufacturing Co., Shanghai, China), an HNYC-2102C incubation oscillator (Tianjin Honour Instrument Co., Tianjin, China), a YXQ-LS-75S11 vertical pressure steam sterilization pot (Shanghai Boxun Industry & commerce Co., Shanghai, China), a GEN10SUV-Vis ultraviolet spectrophotometer (Thermo Fisher Scientific Co., United States).

### 4.4 Extraction and isolation

The dry lichens of *Usnea diffracta* Vain (4.0 Kg; sampling: September 2018) were extracted with 95% v/v EtOH/H_2_O three times for 3 h each time and then 70% v/v EtOH/H_2_O three times for 3 h each time. The extracts were concentrated to afford the crude extract (1.7 kg). The crude extract was dissolved in 5 L pure water to make suspension, and then successively extracted with petroleum ether, dichloromethane, and ethyl acetate. The filtered combined solution of each solvent extraction was evaporated to yield the petroleum ether (101.2 g), CH_2_Cl_2_ (672.5 g), EtOAC (218.1 g), and post-extraction (650.3 g) extracts. The post-extraction extract (650.3 g) was dissolved in pure water and passed through AB-8 macroporous resin (3.5 kg) column, which was divided into insoluble precipitation layer (250.4 g), water layer (149.6 g), 30% ethanol layer (90.3 g), 60% ethanol layer (41.4 g), and 100% ethanol layer (73.5 g).

The petroleum ether extract (2.0 g) was fractionated by column chromatography (CC) using silica gel (300–400 mesh) as the adsorbent. Eluting with a gradient system of petroleum ether–ethyl acetate (100:1–14:1–7:1–3:1) to afford 12 main fractions (Fr1-12) based on TLC investigations. Fraction Fr2 (100:1) was precipitated and crystallized, washed with petroleum ether, and filtered to obtain compound **13** (100.0 mg) as a white acicular crystal. Fraction Fr10 (14:1) was precipitated and crystallized, washed with petroleum ether and dichloromethane, and filtered to obtain compound **14** (40.0 mg) as a white acicular crystal. Fraction Fr7 (25.5 mg, 14:1) was further purified on a Sephadex LH-20 column with dichloromethane-methanol (1:1) to obtain Fr (7-1)–(7-8). Fr7-4 precipitated and crystallized, washed with dichloromethane, and filtered to obtain compound **8** (9.8 mg) as a yellow acicular crystal. Fr7-7 was further purified on a Sephadex LH-20 column with dichloromethane-methanol (1:1) and filtered crystallization to obtain compound **15** (4.7 mg) as a white acicular crystal.

The CH_2_Cl_2_ extract (50.0 g) was fractionated by CC (200–300 mesh) eluting with a gradient system of petroleum ether–ethyl acetate (300:1-1:1) to afford 13 main fractions (Fr1–13), CH_2_Cl_2_-CH_3_OH (10:1-1:1) to afford Fr14. Fraction Fr13 (2.3 g, 1:1) was further purified on a Sephadex LH-20 column with dichloromethane-methanol (1:2) to obtain Fr (13-1)–(13-5). Fr13-4 (0.68 g) was subjected to the 5–50 *μ*m ODS column chromatography and eluted with a gradient of methanol/water (5:95-100:0, v/v) to afford eight fractions Fr (13-4-1)–(13-4-8). The fraction Fr13-4-4 (213.0 mg) was precipitated and crystallized. The crystals (6.0 mg) were filtered and prepared by Waters C18 preparation column (10 mm × 150 mm) in Waters 2535 preparative high performance liquid chromatography (210 nm, 280 nm). Compound **27** (3.2 mg, *t*_*R*_ 10 min) was obtained with acetonitrile/water (65:35, v/v, 2.0 mL/min) as eluent. The remaining fraction was also prepared by the same condition to afford compound **6** (48.2 mg, *t*_*R*_ 77 min), compound **26** (10.3 mg, *t*_*R*_ 71 min), and compound **7** (4.8 mg, *t*_*R*_ 86 min) were obtained with methanol/water (68:32, v/v, 2.0 mL/min) as eluent. The remaining Fr13 were combined into Fr14 and Fr13 + 14 (10.8 g). This fraction was subjected to 5–50 μm ODS column chromatography (11.5 × 3 cm, 38 g) and eluted with a gradient of methanol/water (5:95-100:0, v/v) to afford nine fractions Fr ((13 + 14)-1)-Fr ((13 + 14)-9). Fraction Fr ((13 + 14)-4 (1.2 g) was prepared by Shim Pack PRC (201 nm, 283 nm) with a gradient system of methanol/water (70:30, v/v, 6.0 mL/min) as eluent to afford 12 fractions Fr ((13 + 14)-4)-1-Fr ((13 + 14)-4–12). Fr (13 + 14)-4-11 (*t*_*R*_ 90–95 min, 50.7 mg) was further prepared by Waters C18 preparation column (10 mm × 150 mm) (201 nm, 283 nm) with a gradient system of acetonitrile/water (50:50, v/v, 2.0 mL/min) as eluent to yield compound **2** (*t*_*R*_ 20 min, 25.3 mg) and compound **1** (*t*_*R*_ 26 min, 10.0 mg).

The EtOAC extract (100.0 g) was fractionated by CC (200–300 mesh) eluting with a gradient system of petroleum ether–ethyl acetate (150:1-1:1) to afford 10 main fractions (Fr1-10). Fr2 (110:1, 470.2 mg) was subjected to the CC eluted with petroleum ether to afford Fr (2-1)-(2-4). Fr (2-3) (300.1 mg) was prepared by a YMC column (201 nm, 254 nm) with a gradient system of methanol/water (70%–100% 25 min, 100% 15 min, 100%–70% 10 min, 1.5 mL/min) as eluent to yield Fr (2-3-1)–(2-3–7). Compounds **16** (6.1 mg) and **23** (2.2 mg) were prepared by scraping thin layer silica gel with petroleum ether–ethyl acetate (25:1) from Fr2-3-7 (17.0 mg, *t*_*R*_ 42 min). Fr3 (40:1, 45.0 mg) was successively purified on a CC eluting with a gradient system of petroleum ether–ethyl acetate (120:1) and a YMC column (201 nm, 254 nm) with methanol/water (70%–95% 25 min, 95%–100% 5 min, 100%–70% 10 min, 1.5 mL/min) as eluent to afford compound **9** (3.0 mg, *t*_*R*_ 35 min). Fr4 (25:1) was successively purified on a CC eluting with a gradient system of petroleum ether–ethyl acetate (120:1-30:1) and a YMC column (201 nm, 254 nm) with methanol/water (70%–100% 25 min, 100% 15 min, 100%–70% 10 min, 1.5 mL/min) as eluent to afford compound **17** (8.0 mg, *t*_*R*_ 30 min). Fr4-4 (70:1, 90.0 mg) was prepared by a YMC column under the same condition, then scraper was prepared by thin layer silica gel, and compounds **24** (3.5 mg) and **25** (4.8 mg) were obtained by the expansion of petroleum ether–ethyl acetate 8-1 with 0.01% formic acid. Fr6 (5:1, 430.0 mg) was successively purified on a CC eluting with a gradient system of petroleum ether–ethyl acetate (30:1, 0.01% formic acid) and a YMC column (201 nm, 254 nm) under the same condition to afford compounds **18** (2.8 mg, *t*_*R*_ 13.5 min) and **10** (70.1 mg, *t*_*R*_ 25.5 min). Fr8 (5:1, 7.0 g) was successively purified on a CC, ODS column, and a YMC column (201 nm, 254 nm) with methanol/water (30%–70% 30 min, 70% 15 min, 70%–90% 15 min, 90%–100% 10 min, 100% 10 min, 100%–30% 10 min, 1.5 mL/min) as eluent to afford Fr (8-1)-(8-8). Compound **22** (12.8 mg) was obtained from Fr8-3 (*t*_*R*_ 29–39 min) by a YMC column with 40% elution of methanol/water. Fr8-6 (*t*_*R*_ 51–52 min, 20.0 mg) was further prepared by a YMC column with a gradient system of acetonitrile/water (60:40, v/v) as eluent to yield compound **12** (16.8 mg).

The 60% ethanol layer (2.7 g) was fractionated by 5–50 μm ODS column chromatography and eluted with a gradient of methanol/water (5:95-100:0, v/v) to afford 14 fractions (Fr1-14). Fraction Fr4 (33.1 mg, 30:70) was further purified on a Sephadex LH-20 column with methanol to obtain Fr (4-1)-(4-4). Fr (4-2) was prepared by a YMC column (210 nm, 278 nm) with a gradient system of methanol/water (50:50, v/v, 1.5 mL/min) as eluent to yield four fractions Fr (4-2-1)-(4-2-4). Fr4-2-4 (*t*_*R*_ 17-24 min, 10.9 mg) was further prepared by a YMC semi-preparative column (210 nm, 278 nm) with a gradient system of methanol/0.05% trifluoroacetic acid water (50:50, v/v, 1.5 mL/min) as eluent to yield Fr (4-2-4-1)-(4-2-4-4). Fr4-2-4-3 (*t*_*R*_ 16–21 min, 7.5 mg) was further prepared by a YMC column (210 nm, 278 nm) with a gradient system of acetonitrile/0.07% trifluoroacetic acid water (25:75, v/v, 1.5 mL/min) as eluent to yield compound **5** (*t*_*R*_ 17 min, 3.0 mg) and compound **4** (*t*_*R*_ 18.5 min, 1.5 mg). Fr7 (49.1 mg, 50:50) was subjected to the Sephadex LH-20 column with methanol and CC (200–300 mesh) eluting with a gradient system of petroleum ether–ethyl acetate (15:1) to afford compound **19** (3.6 mg). Fr10 (162.1 mg, 70:30) was purified on a Sephadex LH-20 column with methanol to obtain Fr (10-1)-(10-7). Fr10-5 (13.8 mg) was fractionated by CC (200–300 mesh) eluting with a gradient system of petroleum ether–ethyl acetate (1:1) to afford compound **11** (6.7 mg). Fr10-4 (6.1 mg) was fractionated by CC (200–300 mesh) with petroleum ether–ethyl acetate (50:1) to yield compound **20** (1.7 mg). Fraction Fr14 was successively purified on a Sephadex LH-20 column with methanol and CC (200–300 mesh) eluting with a gradient system of petroleum ether–ethyl acetate (150:1) to afford compound **3** (14.5 mg).

The 30% ethanol layer (75.5 g) was fractionated by CC (200–300 mesh) eluting with a gradient system of dichloromethane-methanol (10:1-1:1) ∼ methanol ∼ methanol-water (1:1) to afford Fr1-6. Fr1 (10:1, 7.5 g) was further purified by CC with dichloromethane-methanol (80:1-8:1) to obtain Fr (1-1)-(1–5). Fr1-2 (80:1, 650.1 mg) was successively purified on a ODS column eluting with methanol/water (5:95-100:0), CC (petroleum ether-ethyl acetate (5:1-1:1) ∼ dichloromethane-methanol (10:1-1:1)) and a YMC column (201 nm, 281 nm) with methanol/water (40:60, 1.5 mL/min) as eluent to afford compound **21** (6.7 mg, *t*_*R*_ 21 min). Fr1-3 (20:1) was subjected to a ODS column eluting with methanol/water (5:95-100:0) to obtain Fr (1-3-1)-(1-3-10). Fr1-3-1 (5:95, 1.58 g) was successively prepared by CC (petroleum ether–ethyl acetate (5:2-3:2) ∼ dichloromethane-methanol (1:1)) and a YMC column with methanol/water (53:47) as eluent to afford compound **28** (5.8 mg, *t*_*R*_ 10 min). The remaining Fr1-3-1 were combined into Fr1-3-2 and Fr1-3-3 (1.4 g). This fraction was successively prepared by Shim Pack PRC (201 nm, 281 nm) with methanol/water (10:90, v/v, 7.0 mL/min) and a YMC column (201 nm, 281 nm) with methanol/water (5:95, 1.5 mL/min) as eluent to afford compounds **29** (3.0 mg, *t*_*R*_ 20 min) and **30** (2.0 mg, *t*_*R*_ 26 min).

The compounds were characterized by CD, UV, IR, HR-ESI-MS, ^1^H NMR, ^13^C NMR data, and 2D NMR.

### 4.5 *In vitro* antimicrobial activity

Referring to the disk diffusion method (Noumi et al., 2018), the inhibition zone of compounds against *Staphylococcus aureus* ATCC6358 and *Escherichia coli* BL21 were determined in order to preliminarily screen their antibacterial activities. RPMI1640 medium microdilution assay (M27-Ed4) (Clinical Laboratory Standard Institute, 2017) issued by the CLSI was performed to identify the minimal inhibitory concentration of compounds against *Candida albicans* ATCC90028. The antimicrobial effect was tested against two pathogenic bacteria including one Gram-positive strain (*S. aureus*) and one Gram-negative bacterial strain (*E. coli*) and fungus strain (*C. albicans*). The detailed protocol can be found in Supplemental Materials.

### 4.6 Anti-AChE assay

The assay of AChE inhibitory activity was determined by the improved spectrophotometric Ellman’s method (Zhu et al., 2019; Saenkham et al., 2020). In brief, 140 μL of 100 mM sodium phosphate buffer (pH 8.0), 20 μL of a solution of AChE (0.05 unit/mL), and 20 μL of test compound were mixed in 96-well plates, and then incubated at 30 °C for 15 min. The reaction was initiated by adding 20 μL of mixture solution of 10 μL 5,5′-dithio-bis-nitrobenzoic acid (DTNB) (10.0 mM) and 10 μL ATCl (7.5 mM), then catalyzed by enzymes at a wavelength of 412 nm and the absorbance was measured after 30 min of incubation at 37 °C. We replaced the test compound with 20 μL of phosphate buffer salt (100 mM) as a blank control. All reactions were performed in 96-well microplates in triplicate. Percentage of inhibition was calculated by the following equation:

$$Inhibition\;activity\left(\%\right)=\frac{Absorbance\;of\;control-Absorbance\;of\;sample}{Absorbance\;of\;control}\times 100.$$

Data were expressed as means ± SD and determined with SPSS 21.0 software.

### 4.7 Molecular docking

Molecular docking studies were generated by using the Discovery Studio 2019 Client program. The 3D crystal structure of recombinant human AChE complexed with galantamine (code ID: 4EY6) was obtained from the protein data bank (PDB) with a resolution of 2.4 Å (Cheung et al., 2012). The 4EY6 protein was optimized through the Prepare Protein function of DS software, and the active pocked was defined by the original ligand molecule. Before molecular docking, the original ligand galantamine was redocked to the AChE active pocket. Default settings were kept for parameters. The structural differences before and after molecular docking were compared. The results showed that the RMSD value was 0.5002 Å, indicating the rationality of the selected docking parameters and scoring function. Therefore, this parameter can be used for subsequent molecular docking research. The small molecules were introduced into DS software, and optimized by Prepare Ligands and Minimization. The CDOCKER module was used to simulate the molecular docking between small molecules and AChE. The docking results were analyzed using Discovery Studio Visualizer. Evaluation of the molecular docking was performed according to ‘–CDOCKEREnergy’ value (Yu et al., 2018).

## 5 Conclusion

In this study, we exhibited how to isolate secondary metabolites from the dry lichen body of *Usnea diffracta* Vain and confirmed their structures by physicochemical properties and various spectral methods. We isolated 30 compounds which belonged to dibenzofurans, multi-substituted benzenes, depsides, organic acids, fatty acids, amino acid, furfural, and nucleoside. It contains five new compounds (**1**–**5**). The results of preliminary screening experiments on antibacterial and antifungal activities revealed that the new compound **3** had certain inhibitory activities against *S. aureus* and *E. coli* with IZ of 6.2 mm and 6.3 mm, depsides **9** and **10** had certain inhibitory effect on *S. aureus* and *C. albicans*. Compound **10** demonstrated the strongest inhibition against *S. aureus* and *C. albicans* with 6.6 mm and 32 μg/ml, respectively. Five isolated dibenzofurans (**1**, **2**, and **6**–**8**) were evaluated for their biological properties toward specific target human AChE involved in AD. This study clearly demonstrated that they could effectively inhibit AChE at 0.3 μmol/ml. The most anti-AChE activity was demonstrated by the lichen secondary metabolite Usneamine H (**2**), which was identified as a new compound. Furthermore, the result of molecular docking was consistent with activity experimental data. This compound exhibited the strongest binding affinity and formed several tight bindings to key residues located in the peripheral active site, acyl pocket, anionic subsite, and the oxyanion hole of AChE. In addition, whether these compounds can be potential antimicrobial agents or drug candidate for the treatment of AD needs further study.

## Data Availability

The original contributions presented in the study are included in the article/Supplemental Material; further inquiries can be directed to the corresponding author.
