# Supplementary material for: Phytochemical composition, antimicrobial activities, and cholinesterase inhibitory properties of the lichen Usnea diffracta Vain
Source: Front Chem. 2023 Jan 6;10:1063645. doi: 10.3389/fchem.2022.1063645 (PMC9853987; doi:10.3389/fchem.2022.1063645)
Supplement: Supplementary file 1 [file DataSheet1.docx]

**Phytochemical Composition, Antimicrobial Activities, and Cholinesterase Inhibitory Properties of the Lichen *Usnea diffracta* Vain**

Yi-Meng Hao ^1^, Yuan-Cong Yan^1^, Qing Zhang ^1^, Bing-Qian Liu ^1^, Chang-Sheng Wu ^2^, and Li‐Ning Wang ^1,^*

^1^ *School of Chinese Materia Medica, Tianjin University of Traditional Chinese Medicine, Tianjin 301617, China;*

^2^ *State Key Laboratory of Microbial Technology, Institute of Microbial Technology, Shandong University, Qingdao 266237, China.*

**^*^ Correspondence**

Corresponding Author:

Li-Ning Wang. E-mail: liningwang@tjutcm.edu.cn. Tel: +86-022 5959 6238.

Content

[1 The spectra of compounds. 1](#_Toc115949085)

[Figure S1. The HR-ESI-MS (CH](#_Toc115949086)_[3](#_Toc115949086)_[OH) of compound](#_Toc115949086) **[1](#_Toc115949086)**[. 1](#_Toc115949086)

[Figure S2. The CD (CH](#_Toc115949087)_[3](#_Toc115949087)_[OH) of compound](#_Toc115949087) **[1](#_Toc115949087)**[. 1](#_Toc115949087)

[Figure S3. The UV (CH](#_Toc115949088)_[3](#_Toc115949088)_[OH) of compound](#_Toc115949088) **[1](#_Toc115949088)**[. 1](#_Toc115949088)

[Figure S4. The IR (CH](#_Toc115949089)_[3](#_Toc115949089)_[OH) of compound](#_Toc115949089) **[1.](#_Toc115949089)** [2](#_Toc115949089)

[Figure S5. The](#_Toc115949090) ^[1](#_Toc115949090)^[H NMR (500 MHz, CD](#_Toc115949090)_[3](#_Toc115949090)_[OD) of compound](#_Toc115949090) **[1](#_Toc115949090)**[. 2](#_Toc115949090)

[Figure S6. The](#_Toc115949091) ^[13](#_Toc115949091)^[C NMR (125 MHz, CD](#_Toc115949091)_[3](#_Toc115949091)_[OD) of compound](#_Toc115949091) **[1](#_Toc115949091)**[. 3](#_Toc115949091)

[Figure S7. The](#_Toc115949092) ^[1](#_Toc115949092)^[H-](#_Toc115949092)^[1](#_Toc115949092)^[H COSY (500 MHz, CD](#_Toc115949092)_[3](#_Toc115949092)_[OD) of compound](#_Toc115949092) **[1](#_Toc115949092)**[. 3](#_Toc115949092)

[Figure S8. The HSQC (500 MHz, CD](#_Toc115949093)_[3](#_Toc115949093)_[OD) of compound](#_Toc115949093) **[1](#_Toc115949093)**[. 4](#_Toc115949093)

[Figure S9. The HMBC (500 MHz, CD](#_Toc115949094)_[3](#_Toc115949094)_[OD) of compound](#_Toc115949094) **[1](#_Toc115949094)**[. 4](#_Toc115949094)

[Figure S10. The NOESY (500 MHz, CD](#_Toc115949095)_[3](#_Toc115949095)_[OD) of compound](#_Toc115949095) **[1](#_Toc115949095)**[. 5](#_Toc115949095)

[Figure S11. The DEPT 135 (125 MHz, CD](#_Toc115949096)_[3](#_Toc115949096)_[OD) of compound](#_Toc115949096) **[1](#_Toc115949096)**[. 5](#_Toc115949096)

[Figure S12. The HR-ESI-MS (CH](#_Toc115949097)_[3](#_Toc115949097)_[OH) of compound](#_Toc115949097) **[2](#_Toc115949097)**[. 6](#_Toc115949097)

[Figure S13. The CD (CH](#_Toc115949098)_[3](#_Toc115949098)_[OH) of compound](#_Toc115949098) **[2](#_Toc115949098)**[. 6](#_Toc115949098)

[Figure S14. The](#_Toc115949099) ^[1](#_Toc115949099)^[H NMR (600 MHz, DMSO-d](#_Toc115949099)_[6](#_Toc115949099)_[) of compound](#_Toc115949099) **[2](#_Toc115949099)**[. 7](#_Toc115949099)

[Figure S15. The](#_Toc115949100) ^[13](#_Toc115949100)^[C NMR (150 MHz, DMSO-d](#_Toc115949100)_[6](#_Toc115949100)_[) of compound](#_Toc115949100) **[2](#_Toc115949100)**[. 7](#_Toc115949100)

[Figure S16. The NOESY (600 MHz, DMSO-d](#_Toc115949101)_[6](#_Toc115949101)_[) of compound](#_Toc115949101) **[2](#_Toc115949101)**[. 7](#_Toc115949101)

[Figure S17. The HR-ESI-MS (CH](#_Toc115949102)_[3](#_Toc115949102)_[OH) of compound](#_Toc115949102) **[3](#_Toc115949102)**[. 8](#_Toc115949102)

[Figure S18. The CD (CH](#_Toc115949103)_[3](#_Toc115949103)_[OH) of compound](#_Toc115949103) **[3](#_Toc115949103)**[. 8](#_Toc115949103)

[Figure S19. The UV (CH](#_Toc115949104)_[3](#_Toc115949104)_[OH) of compound](#_Toc115949104) **[3](#_Toc115949104)**[. 8](#_Toc115949104)

[Figure S20. The IR (CH](#_Toc115949105)_[3](#_Toc115949105)_[OH) of compound](#_Toc115949105) **[3](#_Toc115949105)**[. 9](#_Toc115949105)

[Figure S21. The](#_Toc115949106) ^[1](#_Toc115949106)^[H NMR (500 MHz, CDCl](#_Toc115949106)_[3](#_Toc115949106)_[) of compound](#_Toc115949106) **[3](#_Toc115949106)**[. 9](#_Toc115949106)

[Figure S22. The](#_Toc115949107) ^[13](#_Toc115949107)^[C NMR (125 MHz, CDCl](#_Toc115949107)_[3](#_Toc115949107)_[) of compound](#_Toc115949107) **[3](#_Toc115949107)**[. 10](#_Toc115949107)

[Figure S23. The](#_Toc115949108) ^[1](#_Toc115949108)^[H-](#_Toc115949108)^[1](#_Toc115949108)^[H COSY (500 MHz, CDCl](#_Toc115949108)_[3](#_Toc115949108)_[) of compound](#_Toc115949108) **[3](#_Toc115949108)**[. 10](#_Toc115949108)

[Figure S24. The HSQC (500 MHz, CDCl](#_Toc115949109)_[3](#_Toc115949109)_[) of compound](#_Toc115949109) **[3](#_Toc115949109)**[. 11](#_Toc115949109)

[Figure S25. The HMBC (500 MHz, CDCl](#_Toc115949110)_[3](#_Toc115949110)_[) of compound](#_Toc115949110) **[3](#_Toc115949110)**[. 11](#_Toc115949110)

[Figure S26. The NOESY (500 MHz, CDCl](#_Toc115949111)_[3](#_Toc115949111)_[) of compound](#_Toc115949111) **[3](#_Toc115949111)**[. 12](#_Toc115949111)

[Figure S27. The DEPT (125 MHz, CDCl](#_Toc115949112)_[3](#_Toc115949112)_[) of compound](#_Toc115949112) **[3](#_Toc115949112)**[. 12](#_Toc115949112)

[Figure S28. The HR-ESI-MS (CH](#_Toc115949113)_[3](#_Toc115949113)_[OH) of compound](#_Toc115949113) **[4](#_Toc115949113)**[. 13](#_Toc115949113)

[Figure S29. The CD (CH](#_Toc115949114)_[3](#_Toc115949114)_[OH) of compound](#_Toc115949114) **[4](#_Toc115949114)**[. 13](#_Toc115949114)

[Figure S30. The UV (CH](#_Toc115949115)_[3](#_Toc115949115)_[OH) of compound](#_Toc115949115) **[4](#_Toc115949115)**[. 14](#_Toc115949115)

[Figure S31. The IR (CH](#_Toc115949116)_[3](#_Toc115949116)_[OH) of compound](#_Toc115949116) **[4](#_Toc115949116)**[. 14](#_Toc115949116)

[Figure S32. The](#_Toc115949117) ^[1](#_Toc115949117)^[H NMR (600 MHz, CD](#_Toc115949117)_[3](#_Toc115949117)_[OD) of compound](#_Toc115949117) **[4](#_Toc115949117)**[. 14](#_Toc115949117)

[Figure S33. The](#_Toc115949118) ^[13](#_Toc115949118)^[C NMR (150 MHz, CD](#_Toc115949118)_[3](#_Toc115949118)_[OD) of compound](#_Toc115949118) **[4](#_Toc115949118)**[. 15](#_Toc115949118)

[Figure S34. The](#_Toc115949119) ^[1](#_Toc115949119)^[H-](#_Toc115949119)^[1](#_Toc115949119)^[H COSY (600 MHz, CD](#_Toc115949119)_[3](#_Toc115949119)_[OD) of compound](#_Toc115949119) **[4](#_Toc115949119)**[. 15](#_Toc115949119)

[Figure S35. The HSQC (600 MHz, CD](#_Toc115949120)_[3](#_Toc115949120)_[OD) of compound](#_Toc115949120) **[4](#_Toc115949120)**[. 16](#_Toc115949120)

[Figure S36. The HMBC (600 MHz, CD](#_Toc115949121)_[3](#_Toc115949121)_[OD) of compound](#_Toc115949121) **[4](#_Toc115949121)**[. 16](#_Toc115949121)

[Figure S37. The NOESY (600 MHz, CD](#_Toc115949122)_[3](#_Toc115949122)_[OD) of compound](#_Toc115949122) **[4](#_Toc115949122)**[. 17](#_Toc115949122)

[Figure S38. The DEPT 135 (150 MHz, CD](#_Toc115949123)_[3](#_Toc115949123)_[OD) of compound](#_Toc115949123) **[4](#_Toc115949123)**[. 17](#_Toc115949123)

[Figure S39. The HR-ESI-MS (CH](#_Toc115949124)_[3](#_Toc115949124)_[OH) of compound](#_Toc115949124) **[5](#_Toc115949124)**[. 18](#_Toc115949124)

[Figure S40. The](#_Toc115949125) ^[1](#_Toc115949125)^[H NMR (600 MHz, CD](#_Toc115949125)_[3](#_Toc115949125)_[OD) of compound](#_Toc115949125) **[5](#_Toc115949125)**[. 18](#_Toc115949125)

[Figure S41. The](#_Toc115949126) ^[13](#_Toc115949126)^[C NMR (150 MHz, CD](#_Toc115949126)_[3](#_Toc115949126)_[OD) of compound](#_Toc115949126) **[5](#_Toc115949126)**[. 19](#_Toc115949126)

[Figure S42. The](#_Toc115949127) ^[1](#_Toc115949127)^[H-](#_Toc115949127)^[1](#_Toc115949127)^[H COSY (600 MHz, CD](#_Toc115949127)_[3](#_Toc115949127)_[OD) of compound](#_Toc115949127) **[5](#_Toc115949127)**[. 19](#_Toc115949127)

[Figure S43. The HSQC (600 MHz, CD](#_Toc115949128)_[3](#_Toc115949128)_[OD) of compound](#_Toc115949128) **[5](#_Toc115949128)**[. 20](#_Toc115949128)

[Figure S44. The HMBC (600 MHz, CD](#_Toc115949129)_[3](#_Toc115949129)_[OD) of compound](#_Toc115949129) **[5](#_Toc115949129)**[. 20](#_Toc115949129)

[Figure S45. The NOESY (600 MHz, CD](#_Toc115949130)_[3](#_Toc115949130)_[OD) of compound](#_Toc115949130) **[5](#_Toc115949130)**[. 21](#_Toc115949130)

[Figure S46. The DEPT 135 (150 MHz, CD](#_Toc115949131)_[3](#_Toc115949131)_[OD) of compound](#_Toc115949131) **[5](#_Toc115949131)**[. 21](#_Toc115949131)

[2 The detailed protocol of antimicrobial activity assay 21](#_Toc115949133)

[2.1 Disk-Diffusion Assay for](#_Toc115949134) *[S. aureus](#_Toc115949134)* [and](#_Toc115949134) *[E. coli](#_Toc115949134)* [21](#_Toc115949134)

[2.1.1 Preparation of bacterial suspension 22](#_Toc115949135)

[2.1.2 Determination of zone of inhibition (IZ) 22](#_Toc115949136)

[2.2 Microdilution Assay for](#_Toc115949137) *[C. albicans](#_Toc115949137)* [22](#_Toc115949137)

# The spectra of compounds.


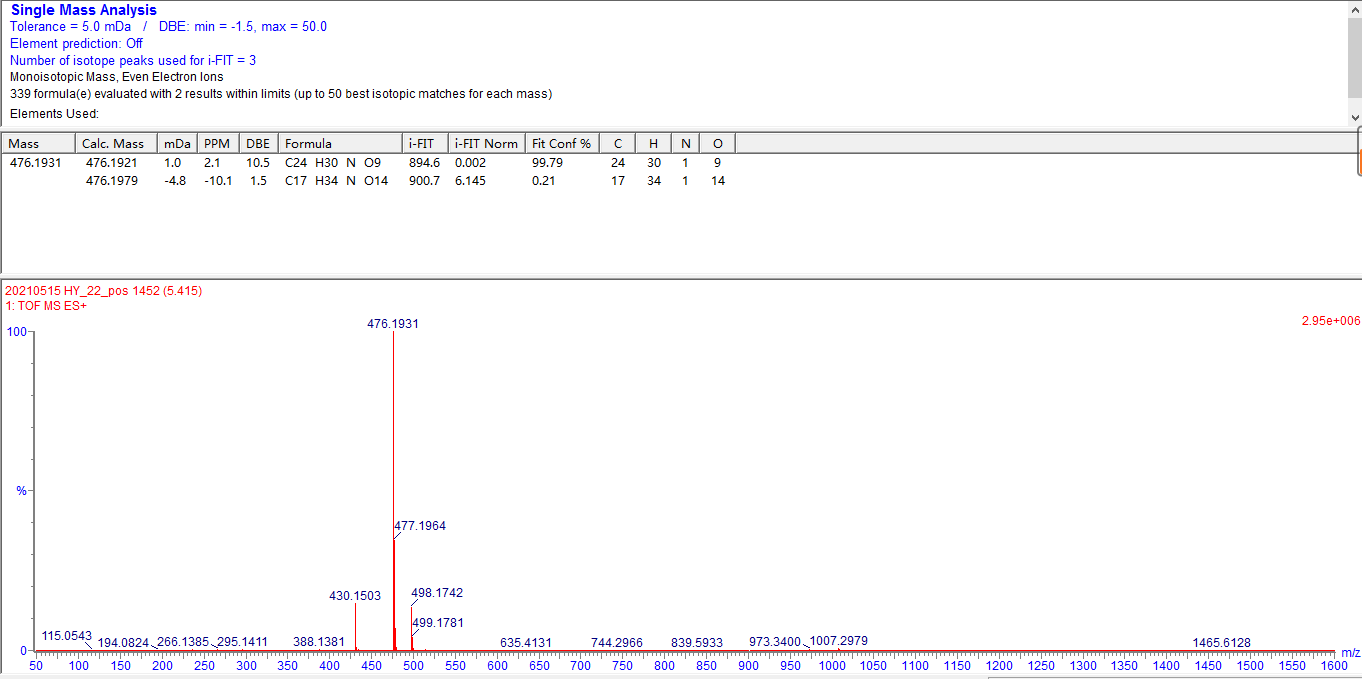


Figure S1. The HR-ESI-MS (CH_3_OH) of compound 1.


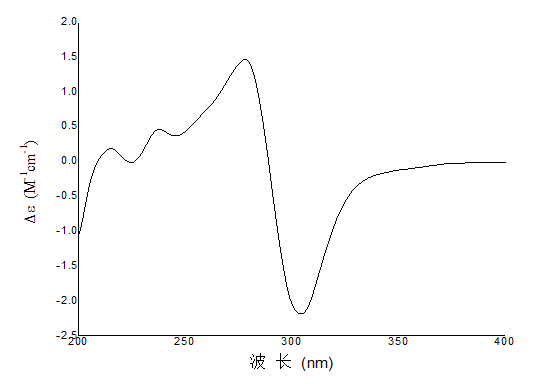


Figure S2. The CD (CH_3_OH) of compound 1.


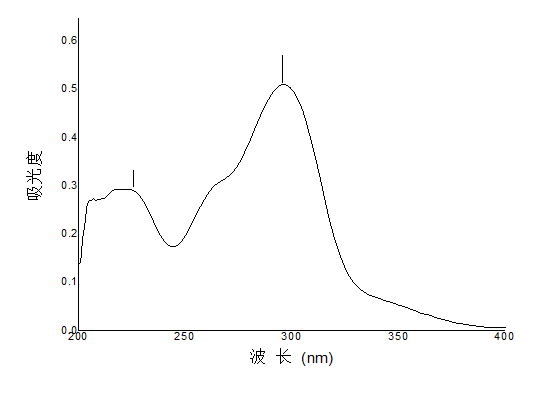


Figure S3. The UV (CH_3_OH) of compound 1.


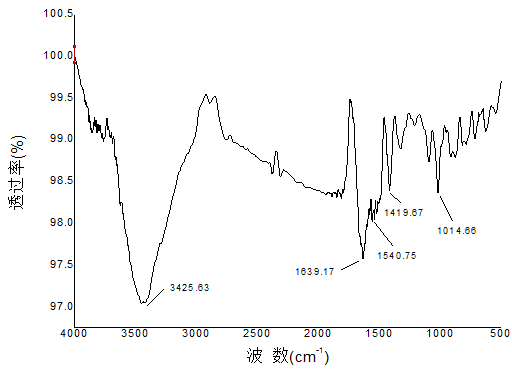


Figure S4. The IR (CH_3_OH) of compound 1.


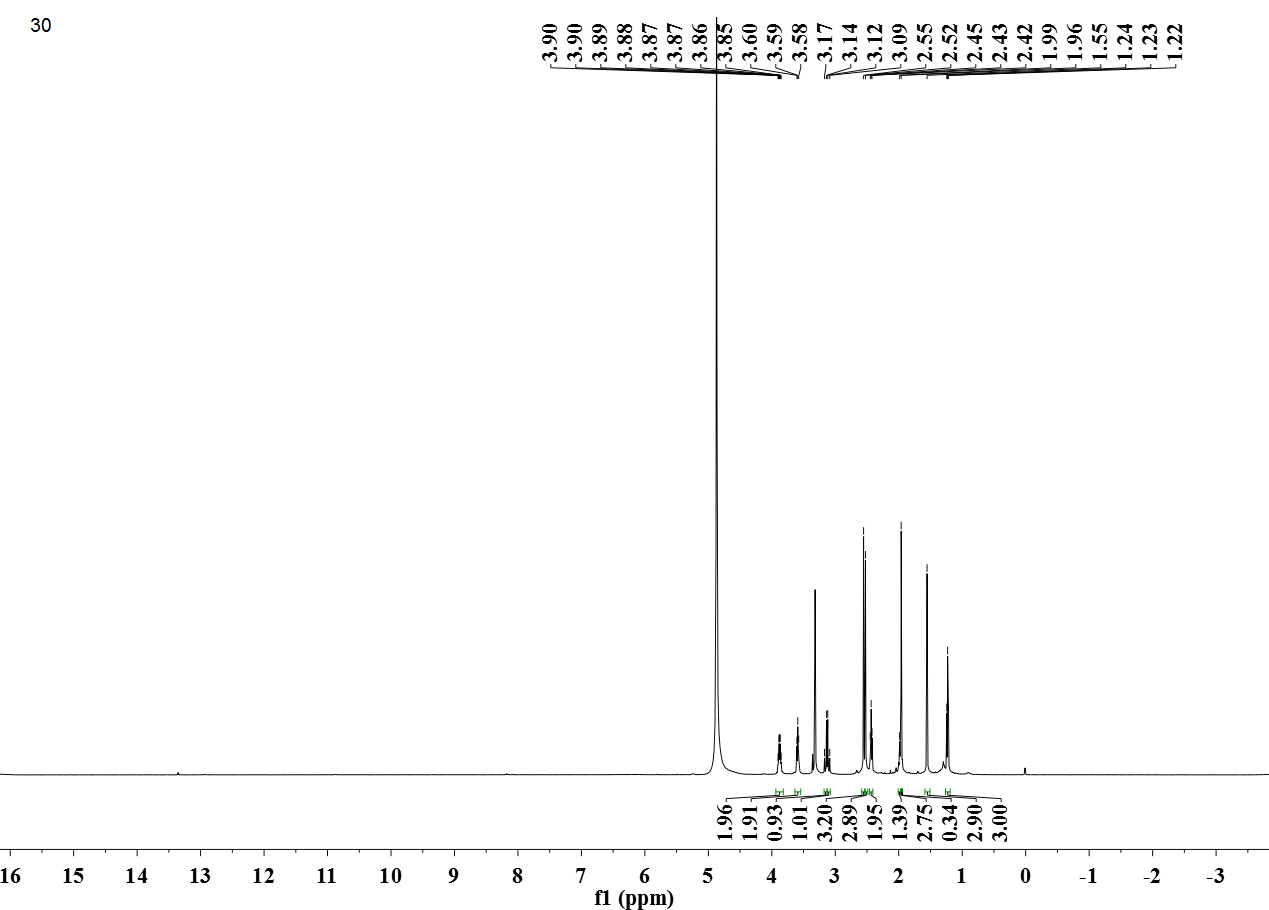


Figure S5. The ^1^H NMR (500 MHz, CD_3_OD) of compound 1.


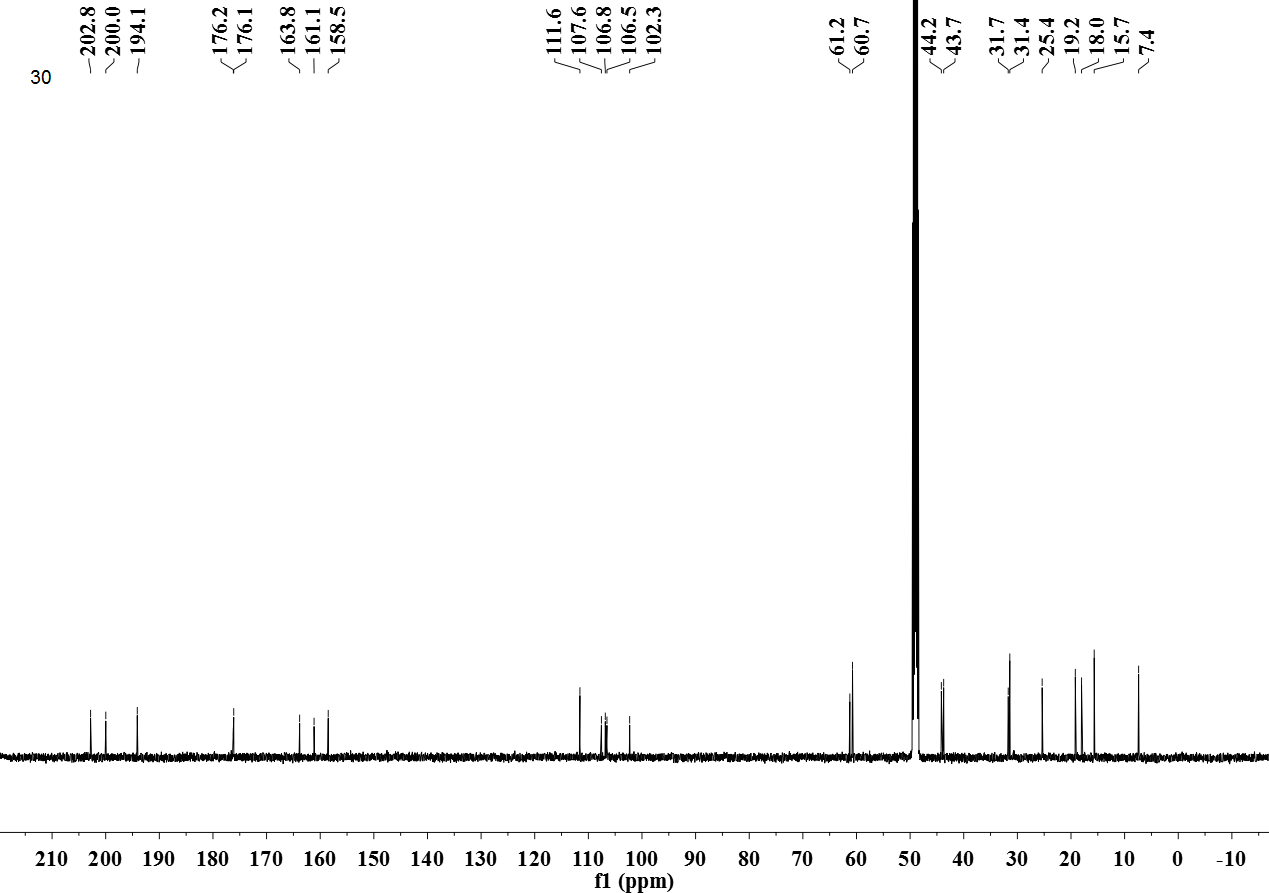


Figure S6. The ^13^C NMR (125 MHz, CD_3_OD) of compound 1.


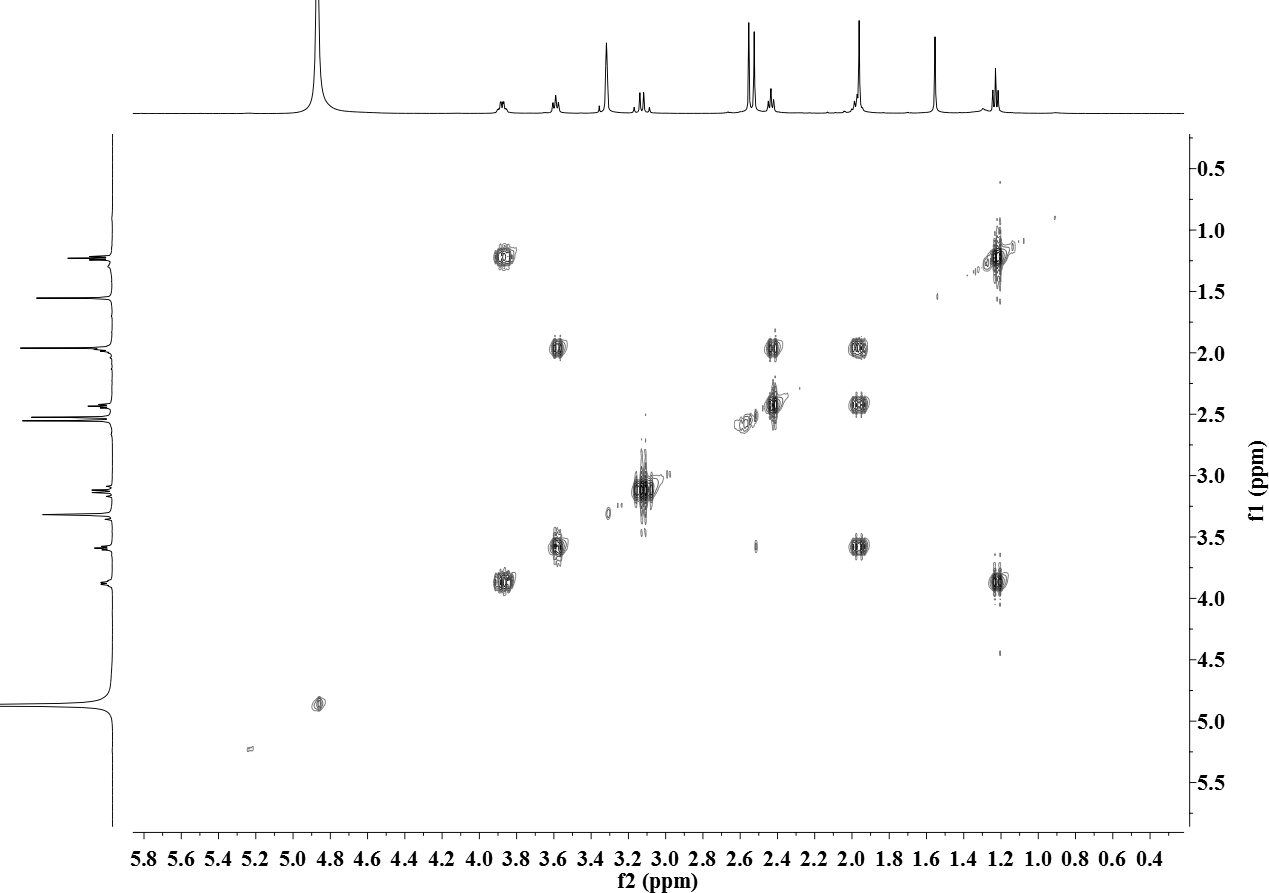


Figure S7. The ^1^H-^1^H COSY (500 MHz, CD_3_OD) of compound 1.


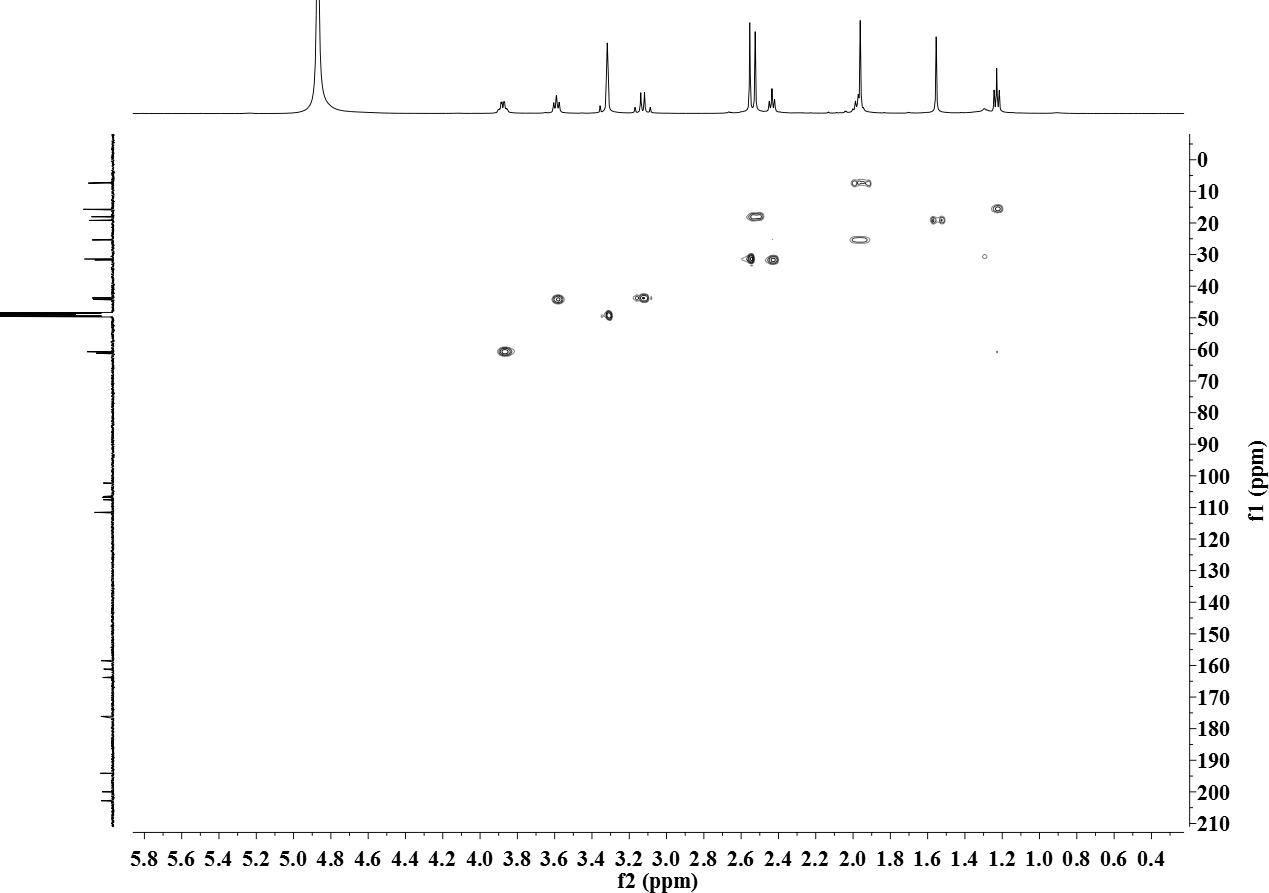


Figure S8. The HSQC (500 MHz, CD_3_OD) of compound 1.


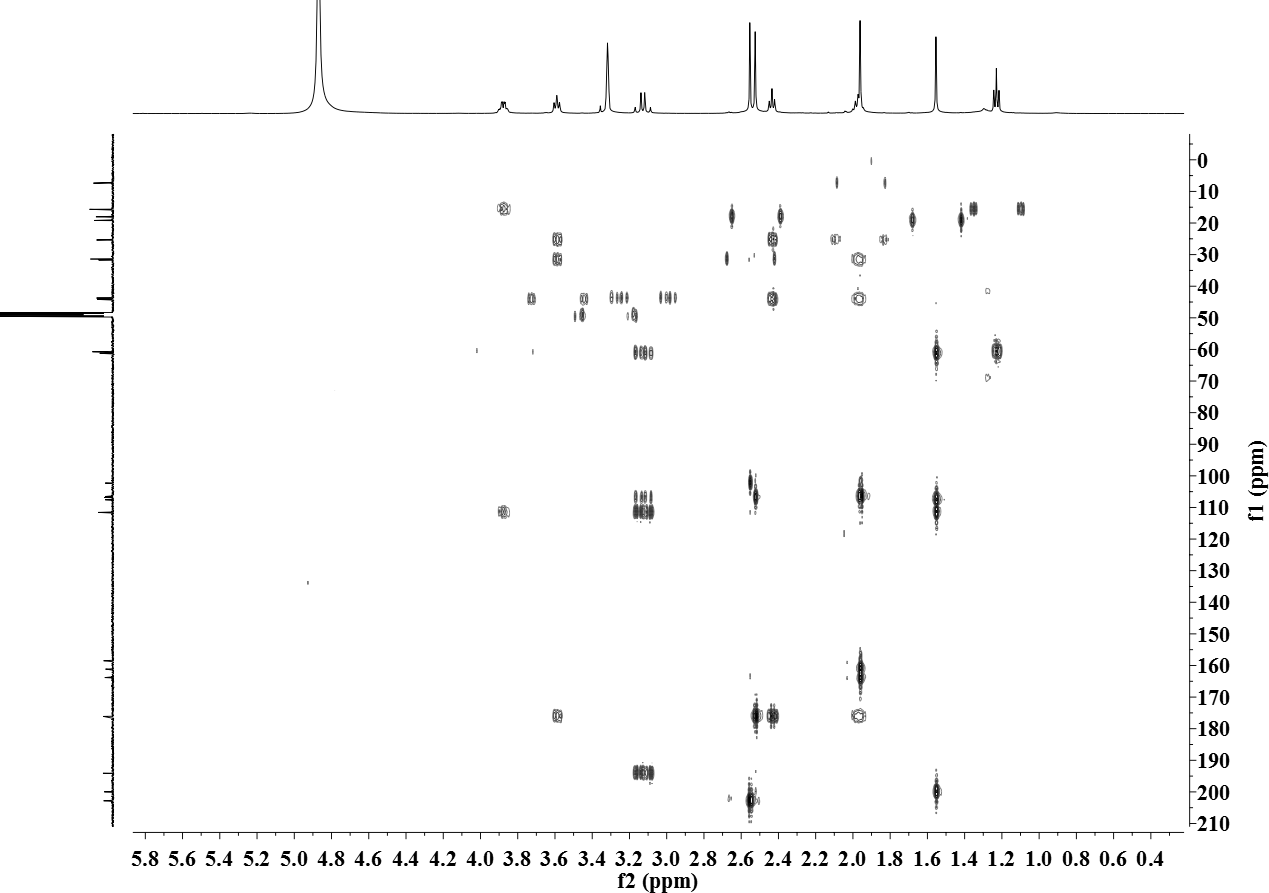


Figure S9. The HMBC (500 MHz, CD_3_OD) of compound 1.


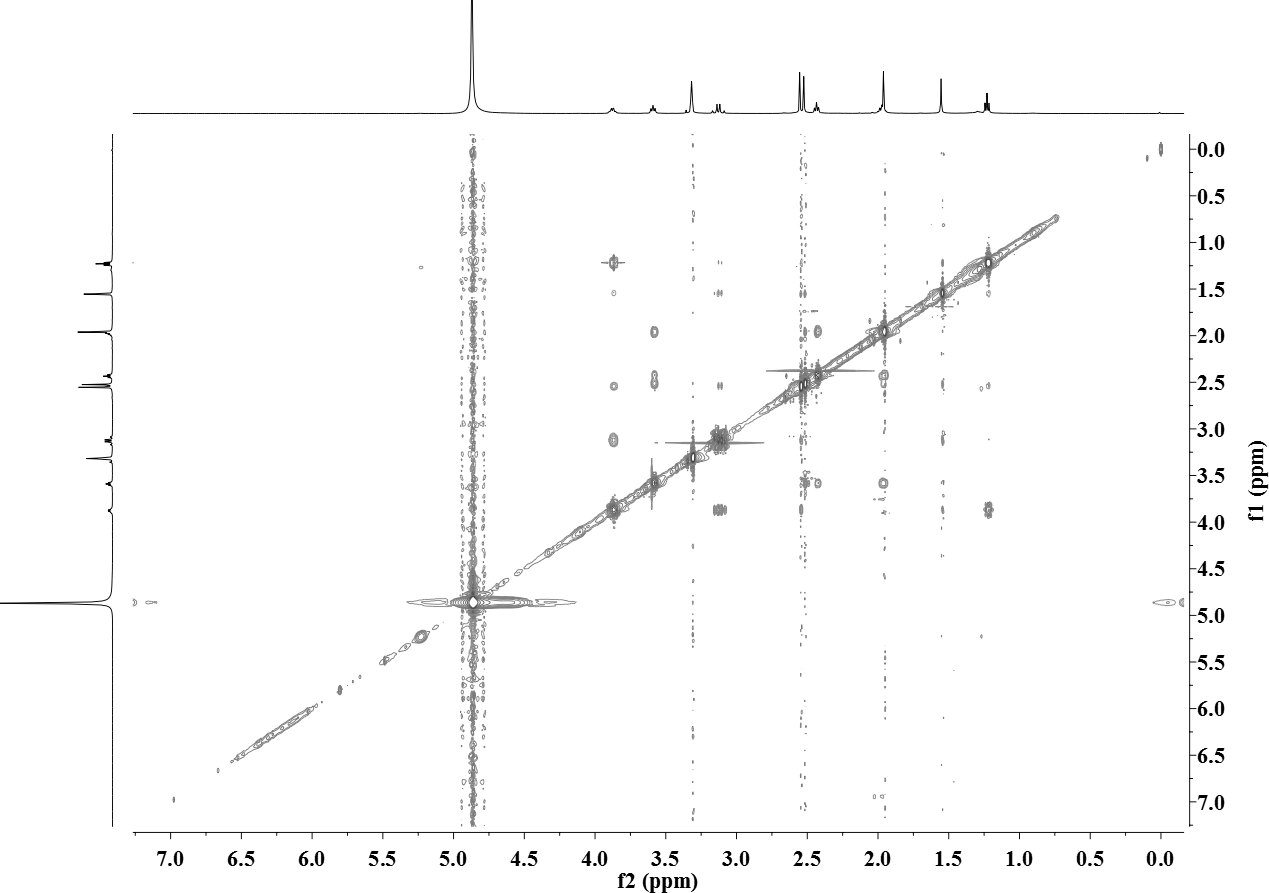


Figure S10. The NOESY (500 MHz, CD_3_OD) of compound 1.


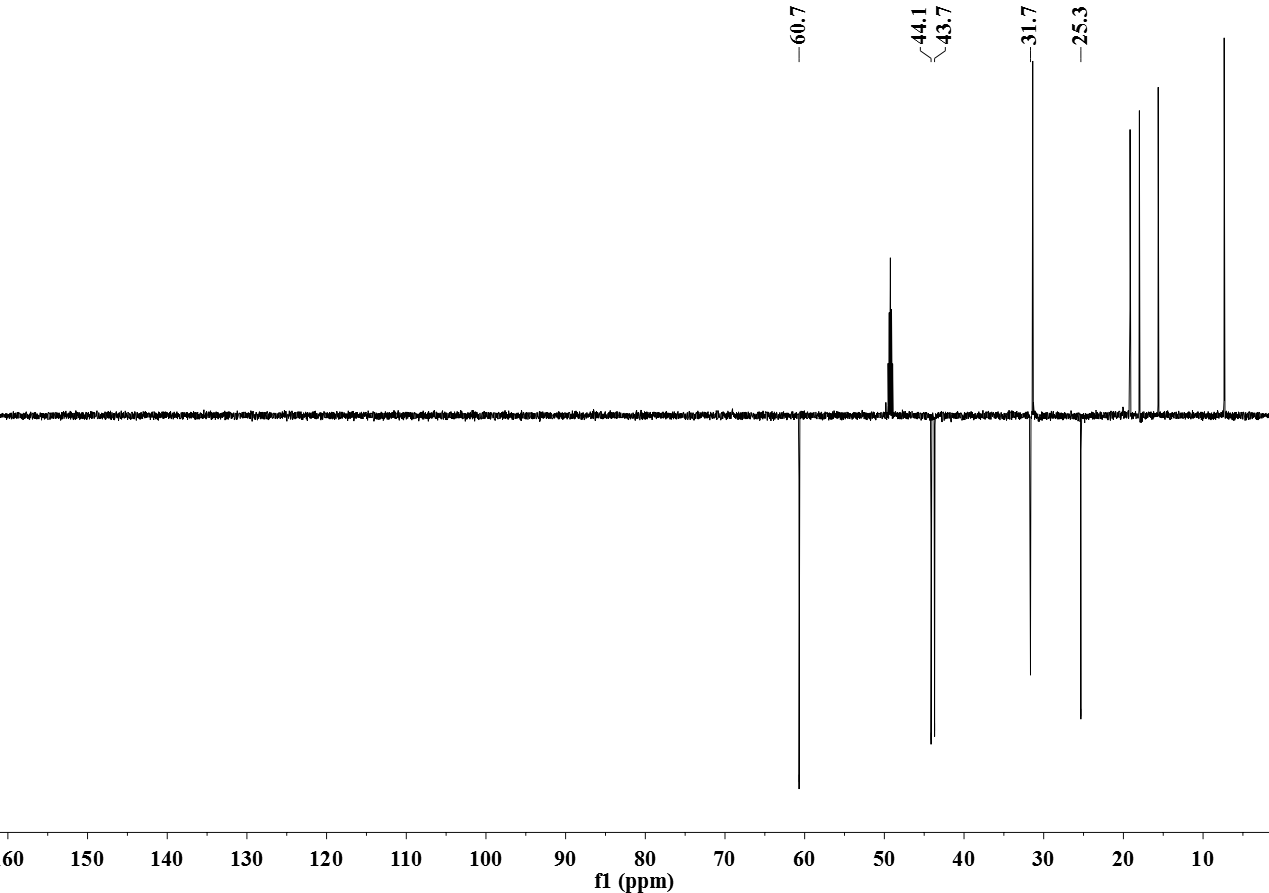


Figure S11. The DEPT 135 (125 MHz, CD_3_OD) of compound 1.


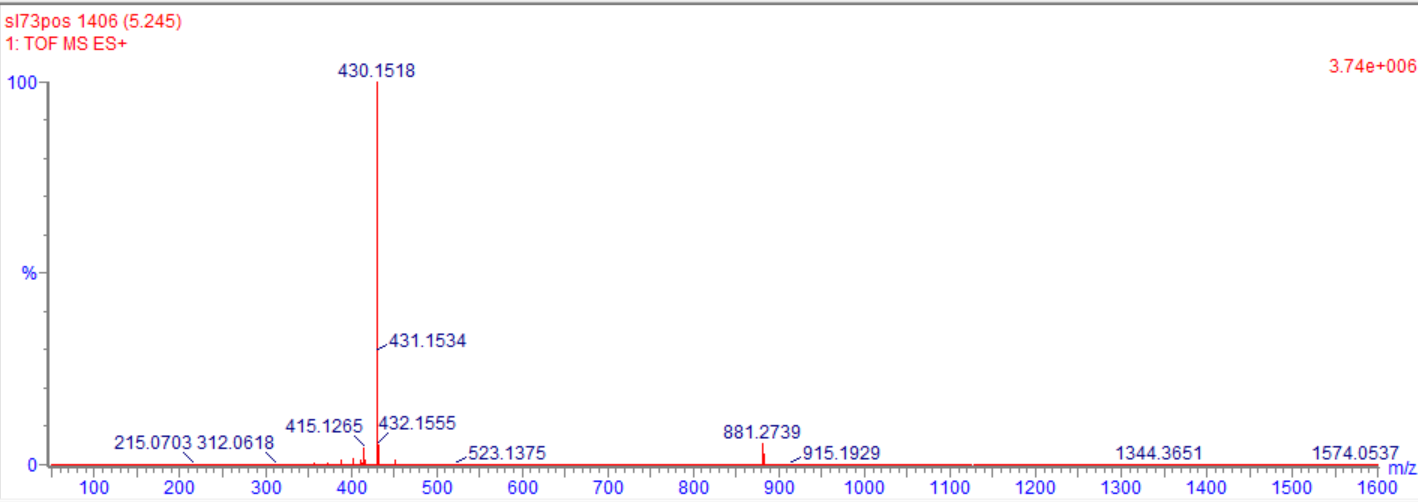


Figure S12. The HR-ESI-MS (CH_3_OH) of compound 2.


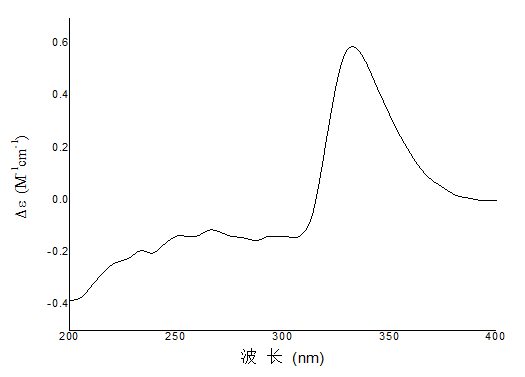


Figure S13. The CD (CH_3_OH) of compound 2.


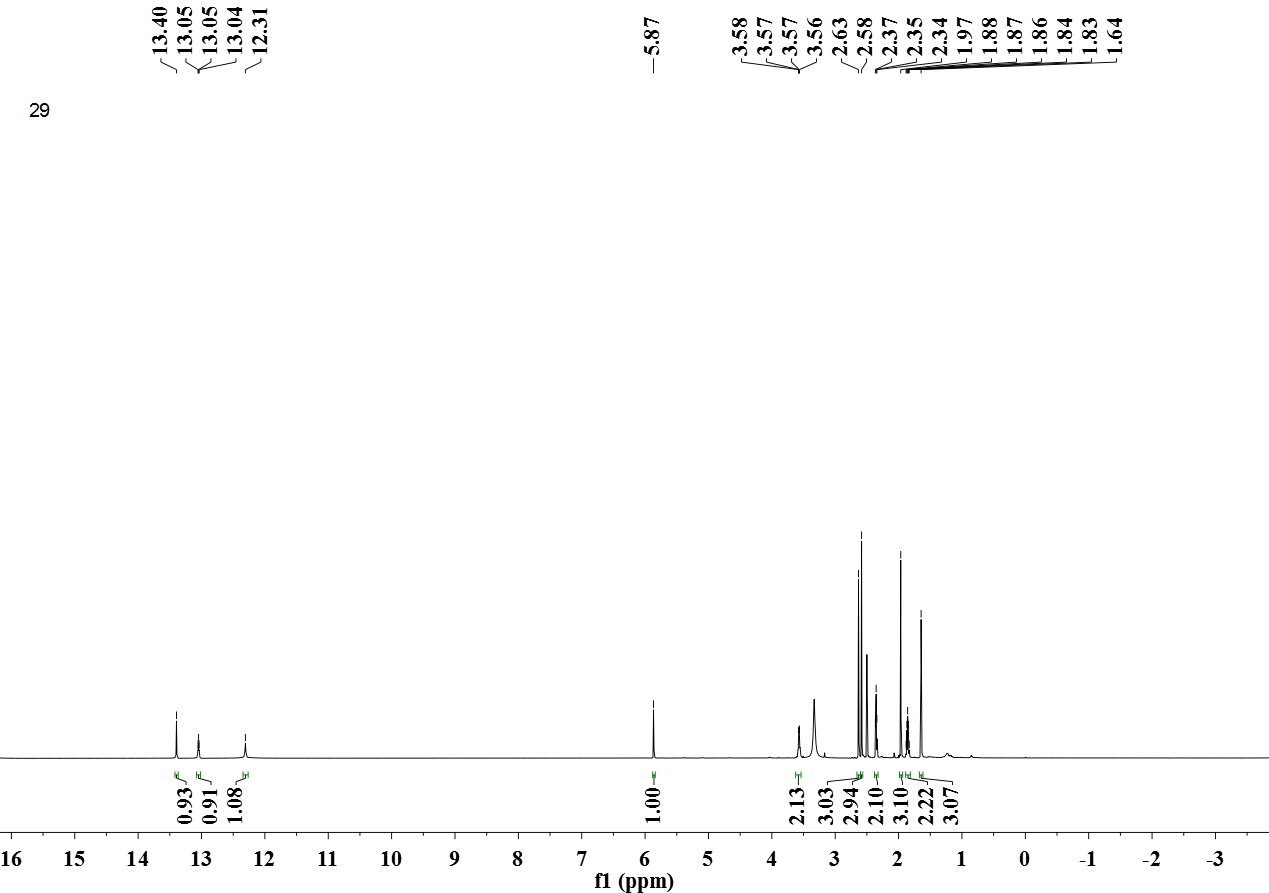


Figure S14. The ^1^H NMR (600 MHz, DMSO-d_6_) of compound 2.


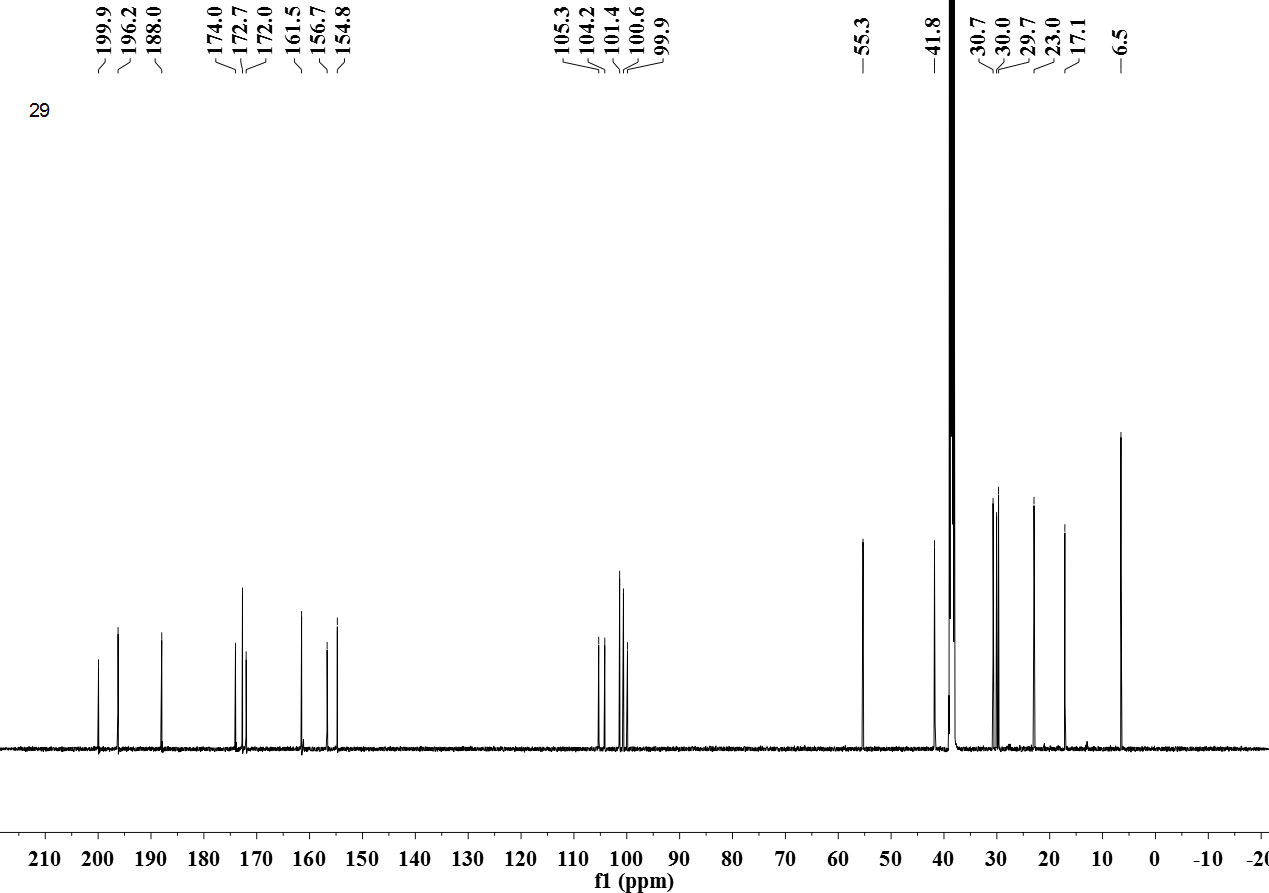


Figure S15. The ^13^C NMR (150 MHz, DMSO-d_6_) of compound 2.


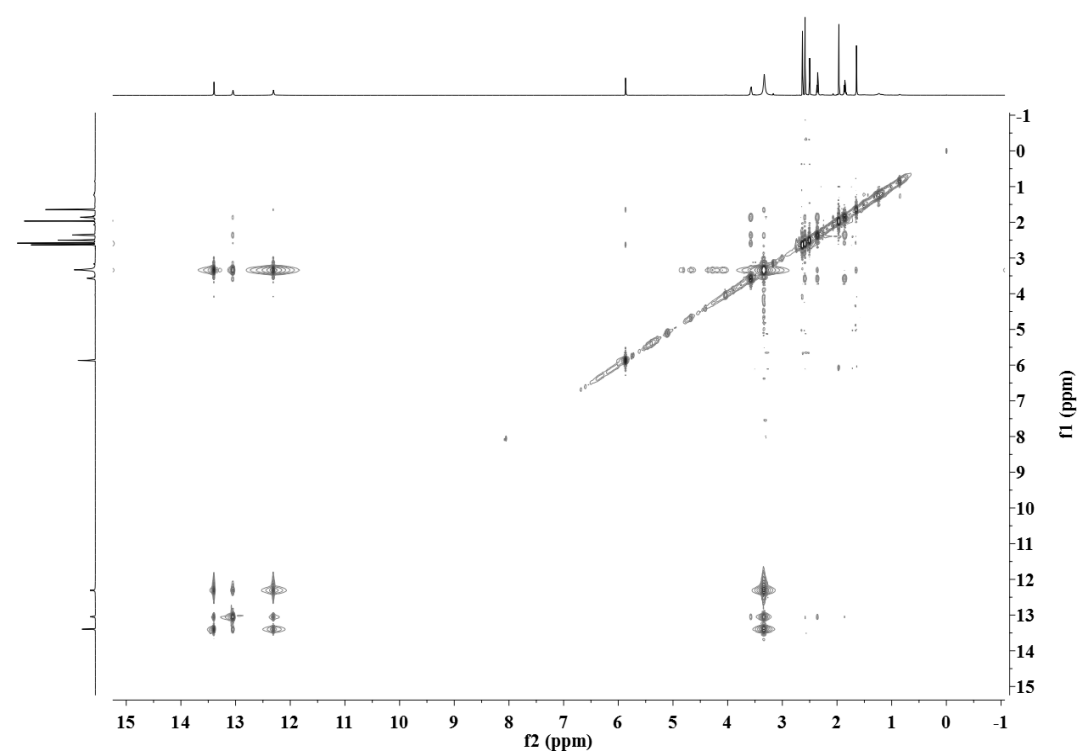


Figure S16. The NOESY (600 MHz, DMSO-d_6_) of compound 2.

Figure S17. The HR-ESI-MS (CH_3_OH) of compound 3.

Figure S18. The CD (CH_3_OH) of compound 3.

Figure S19. The UV (CH_3_OH) of compound 3.


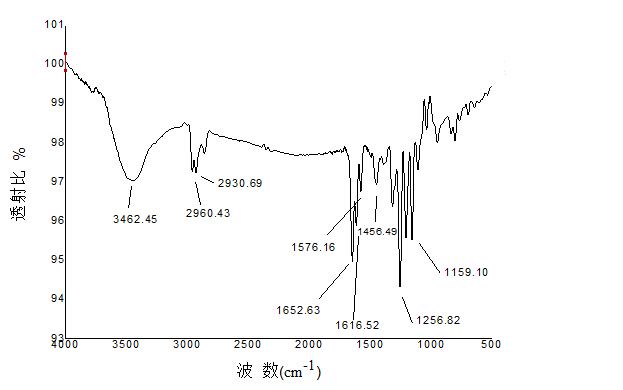


Figure S20. The IR (CH_3_OH) of compound 3.

Figure S21. The ^1^H NMR (500 MHz, CDCl_3_) of compound 3.

Figure S22. The ^13^C NMR (125 MHz, CDCl_3_) of compound 3.


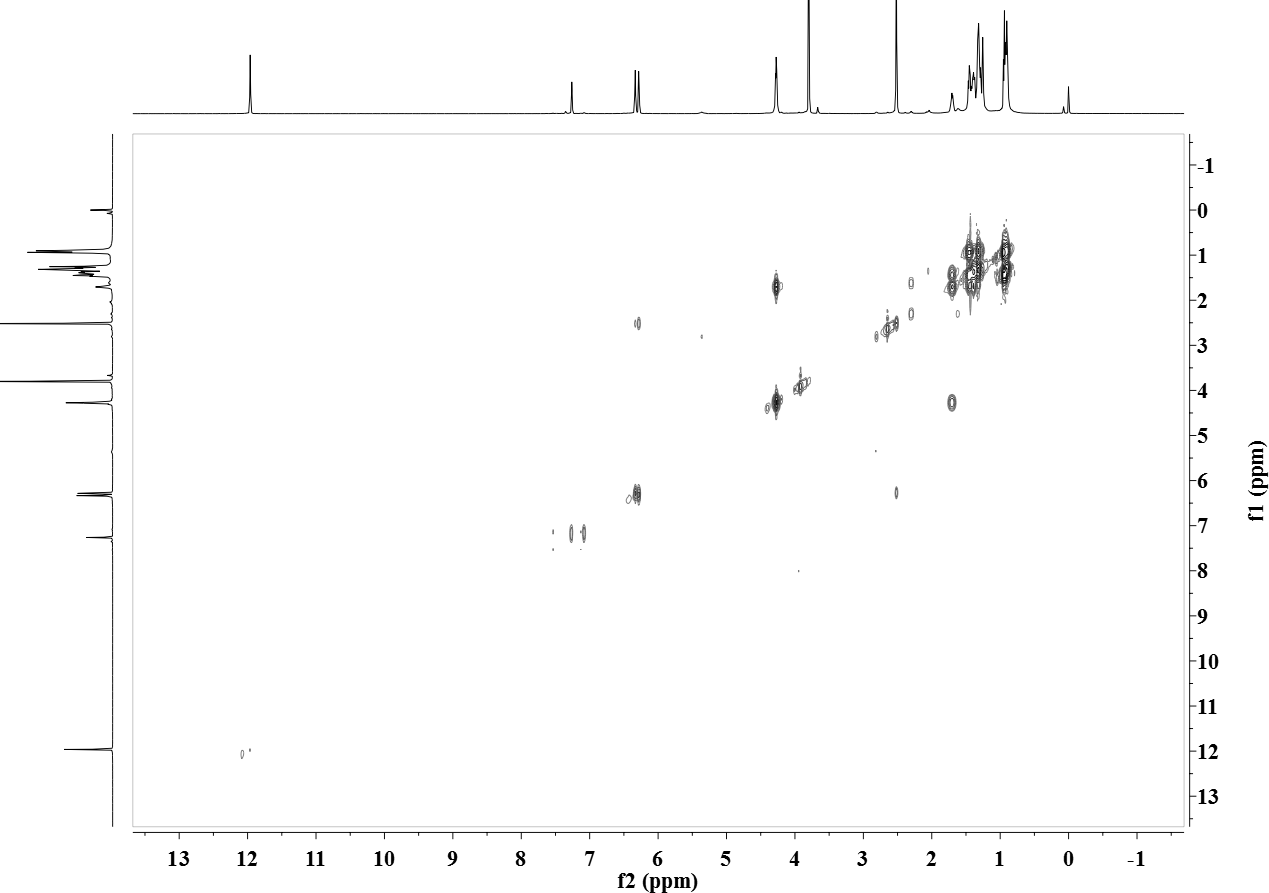


Figure S23. The ^1^H-^1^H COSY (500 MHz, CDCl_3_) of compound 3.

Figure S24. The HSQC (500 MHz, CDCl_3_) of compound 3.

Figure S25. The HMBC (500 MHz, CDCl_3_) of compound 3.


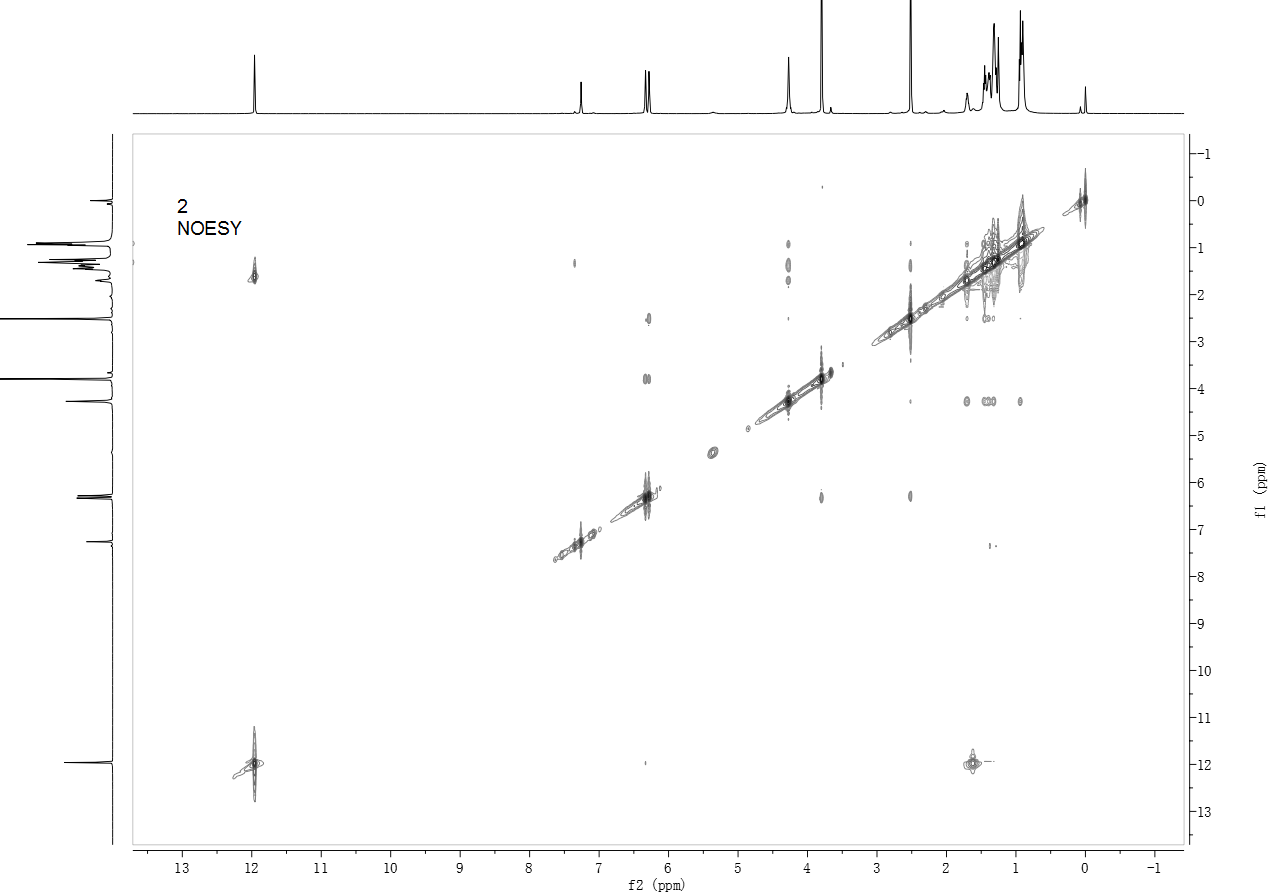


Figure S26. The NOESY (500 MHz, CDCl_3_) of compound 3.

Figure S27. The DEPT (125 MHz, CDCl_3_) of compound 3.

Figure S28. The HR-ESI-MS (CH_3_OH) of compound 4.

Figure S29. The CD (CH_3_OH) of compound 4.

Figure S30. The UV (CH_3_OH) of compound 4.


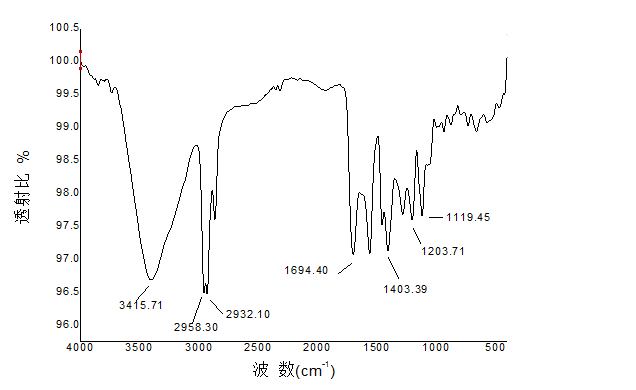


Figure S31. The IR (CH_3_OH) of compound 4.

Figure S32. The ^1^H NMR (600 MHz, CD_3_OD) of compound 4.

Figure S33. The ^13^C NMR (150 MHz, CD_3_OD) of compound 4.

Figure S34. The ^1^H-^1^H COSY (600 MHz, CD_3_OD) of compound 4.

Figure S35. The HSQC (600 MHz, CD_3_OD) of compound 4.

Figure S36. The HMBC (600 MHz, CD_3_OD) of compound 4.


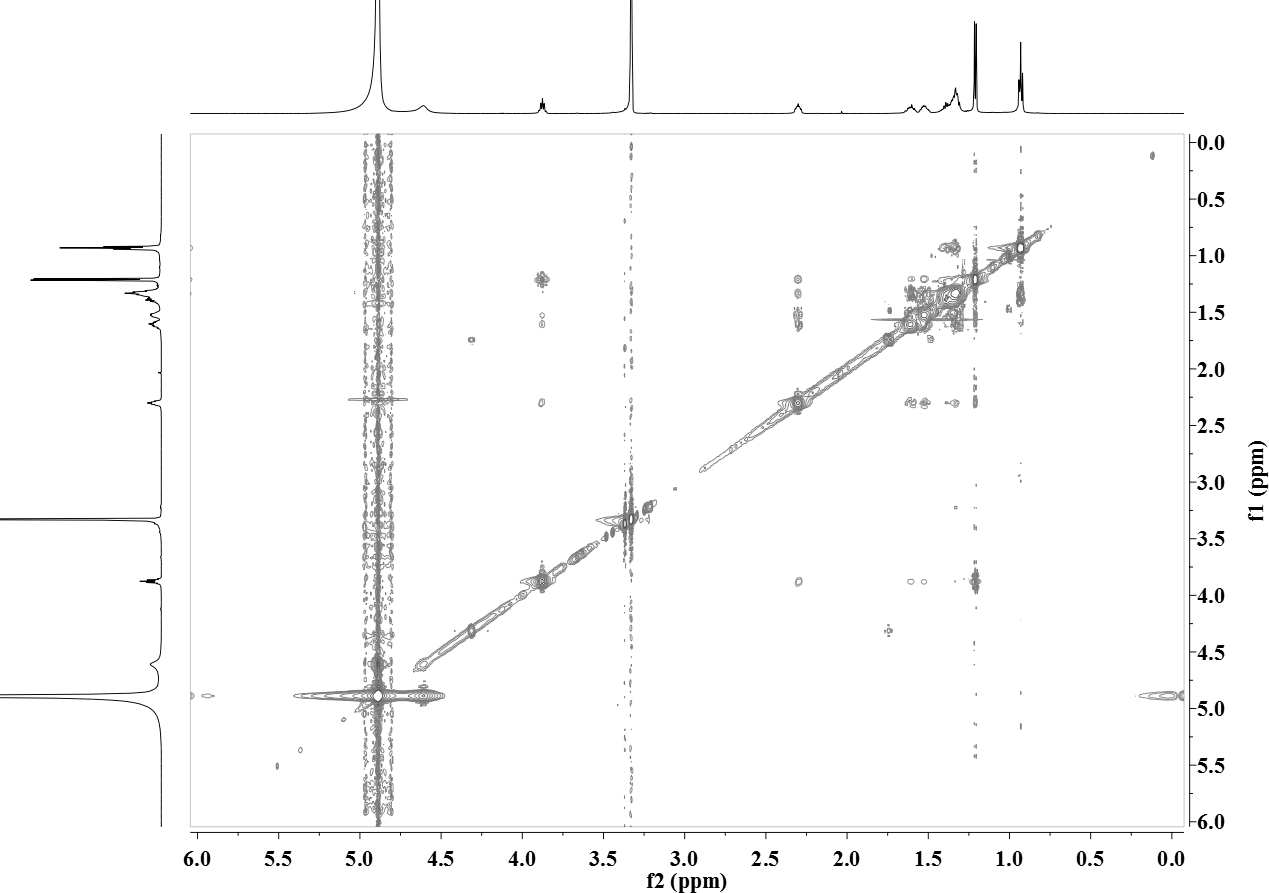


Figure S37. The NOESY (600 MHz, CD_3_OD) of compound 4.

Figure S38. The DEPT 135 (150 MHz, CD_3_OD) of compound 4.


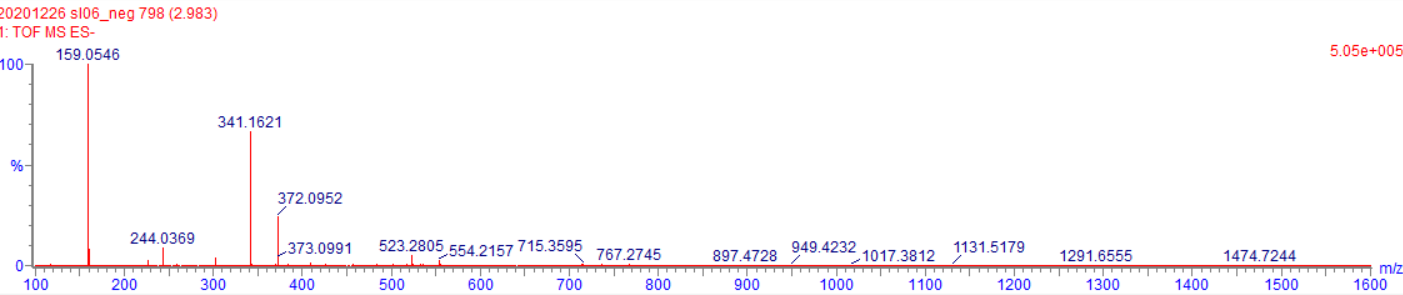


Figure S39. The HR-ESI-MS (CH_3_OH) of compound 5.

Figure S40. The ^1^H NMR (600 MHz, CD_3_OD) of compound 5.

Figure S41. The ^13^C NMR (150 MHz, CD_3_OD) of compound 5.


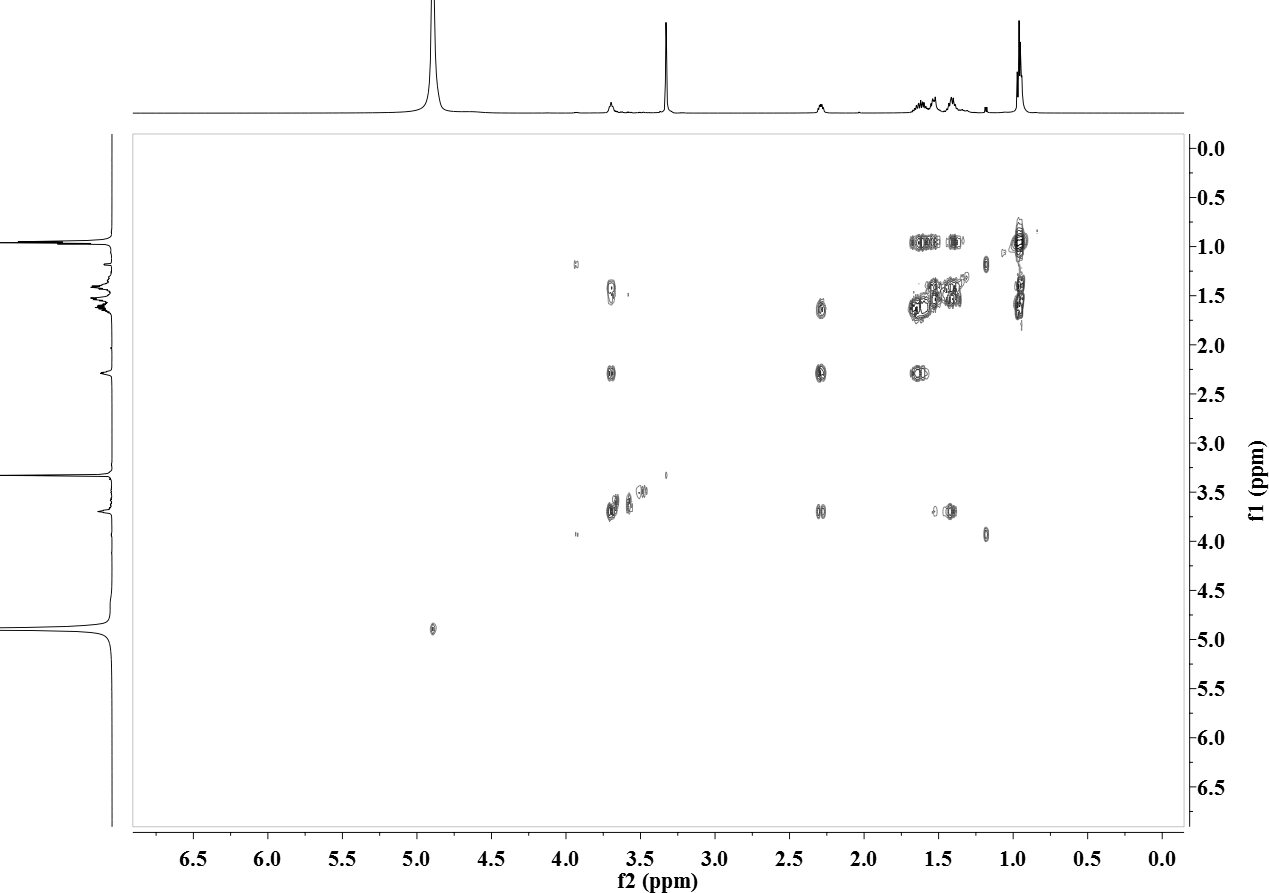


Figure S42. The ^1^H-^1^H COSY (600 MHz, CD_3_OD) of compound 5.

Figure S43. The HSQC (600 MHz, CD_3_OD) of compound 5.

Figure S44. The HMBC (600 MHz, CD_3_OD) of compound 5.


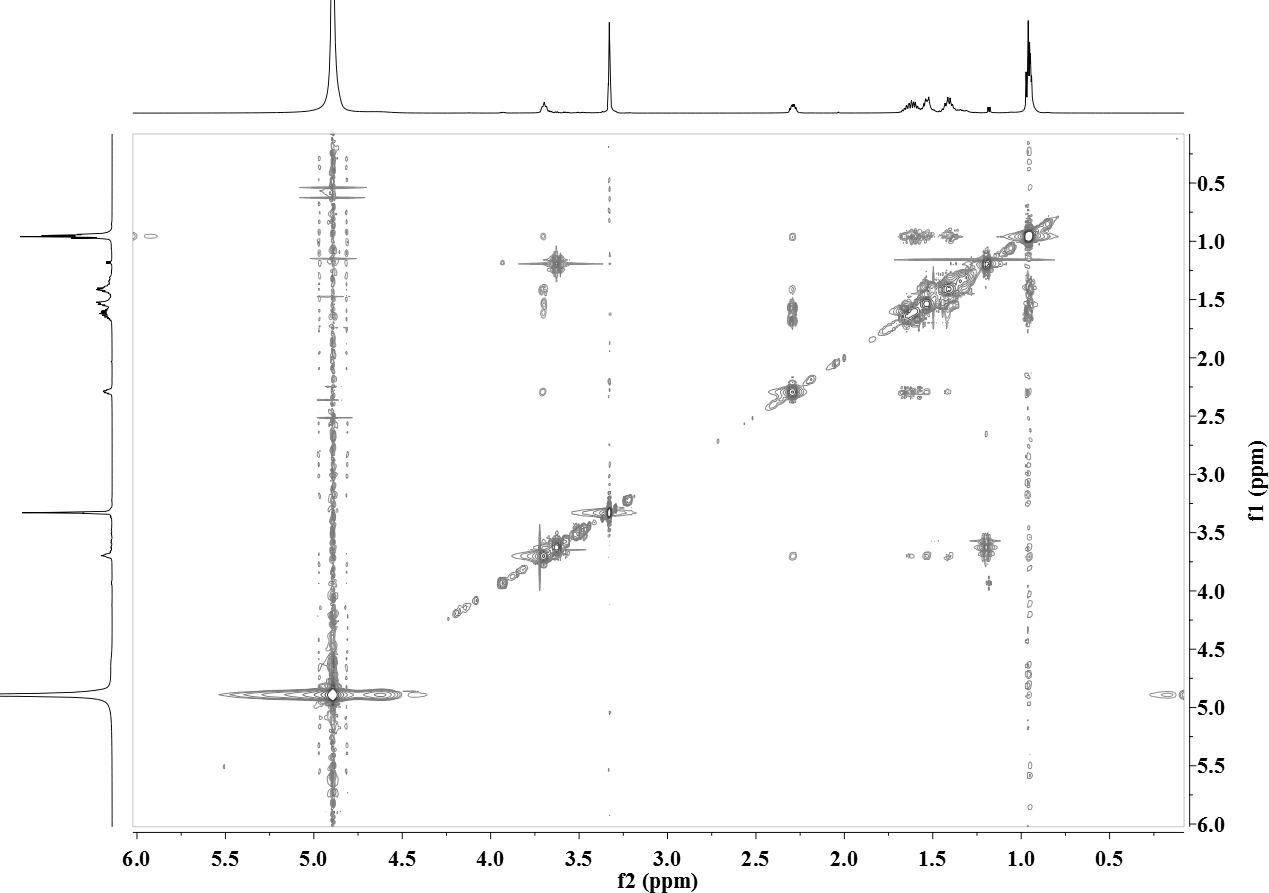


Figure S45. The NOESY (600 MHz, CD_3_OD) of compound 5.

Figure S46. The DEPT 135 (150 MHz, CD_3_OD) of compound 5.


# The detailed protocol of antimicrobial activity assay

## Disk-Diffusion Assay for *S. aureus* and *E. coli*

### Preparation of bacterial suspension

Strains *S.aureus* and *E. coli* were expanded and cultured in BHI and LB liquid medium with sterilized toothpick dipped in seed preservation solution at 37℃ for 24 hours (180 r/min). The plate marking method was inoculated in the corresponding solid medium and cultured at 37℃ for 18 h. A single clone was taken out with a sterilized toothpick, inoculated into the liquid medium and cultured overnight in a constant temperature shaker. The absorbance value (OD_600_) was measured according to the standard of 1 × 10^8^ CFU/mL per 0.1 OD_600_. The bacteria solution was diluted with freshly sterilized BHI liquid medium to the absorbance value of 0.5.

### Determination of zone of inhibition (IZ)

In a sterile ultra-clean workbench, the bacterial suspension was taken twice, and spread 500 µL each time in sterilized solid medium to make a bacteria-containing plate. The sample disk (diameter 6 mm) containing 25 µL concentration of 128.0 µg/mL was equidistantly attached to the bacterial plate, DMSO disk was used as a negative control, and Gentamicin sulfate as a positive control. Incubate at 37℃ for 18 h, each sample was operated in parallel for 3 times, the diameter of the inhibition zone was measured, and the results were averaged.

## Microdilution Assay for *C. albicans*

Minimum inhibitory concentration (MIC) values were determined according to the guidelines of the Clinical and Laboratory Standards of the Institute (M27-Ed4). Briefly, 10 µL of *C. albicans* solution was cultured in Sabouraud solid medium (SDA) (35℃, 24 h). Single clones were picked and cultured again. 2-3 fresh clones were put into 1.3 mL 0.85% sterile saline, and then diluted continuously for 10 times to obtain 1~5×10^3^ CFU/ml. The compounds dissolved in dimethylsulfoxide (DMSO) with a high concentration about 512 µg/mL. Serial two-fold dilutions were prepared in 96-wells plate with RPMI1640 media. In fact, 25 µL aliquot from the stock solution (512 µg/mL) was added to the first well containing 175 µL of the RPMI1640 media. Then, a serial two-fold dilutions was prepared by transferring 100 µL from the first well into the six consecutive wells which contained 100 µL of the RPMI1640 media to yield final concentrations of constituents of 64, 32, 16, 8, 4, 2 and 1 µg/mL respectively. The last well containing 100 µL of RPMI1640 media and 100 µL of the inoculum on each strain was used as the negative control. Finally, 100 µL of the inoculum of each microorganism was added to the wells with a final volume about 200 µL in each well. Fluconazol was used as positive control. The 96-well plates were incubated for 24 h at 35℃. The MIC was defined as the lowest concentration of drugs or compounds that completely inhibited the growth of *C. albicans*.
